# The involvement of TRPV1 in the apoptosis of spermatogenic cells in the testis of mice with cryptorchidism

**DOI:** 10.1038/s41420-025-02447-3

**Published:** 2025-04-03

**Authors:** Yanqiu Zhao, Jinhua Wei, Pang Cheng, Junxian Ma, Bo Liu, Mingxiang Xiong, Ting Gao, Jingqi Yao, Tianchen Sun, Zhen Li

**Affiliations:** 1 Department of Human Anatomy, Histology and Embryology, Air Force Medical University, Xi’an, China; 2 The Air Force Hospital of Central Theater of PLA, Datong, China

**Keywords:** Urogenital reproductive disorders, Experimental models of disease, Disease model

## Abstract

Cryptorchidism is associated with an increased risk of male infertility and testicular cancer. Persistent exposure to high temperature in cryptorchidism can lead to the apoptosis of spermatogenic cells. Transient receptor potential vanilloid 1 (TRPV1), a thermosensitive cation channel, has been found to have differential effects on various apoptosis processes. However, whether TRPV1 is involved in spermatogenic cell apoptosis induced by cryptorchidism remains unclear. Herein, we first observed the expression pattern of TRPV1 in the testes of mice with experimental cryptorchidism, and then investigated the role and mechanism of TRPV1 in spermatogenic cell apoptosis by using *Trpv1*^*−/−*^ mice. The results showed that TRPV1 was highly expressed on the membrane of spermatocytes in mouse testis, and the expression increased significantly in the testis of mice with experimental cryptorchidism. After the operation, *Trpv1*^*−/−*^ mice exhibited less reproductive damage and fewer spermatogenic cell apoptosis compared to the wild-type (WT) mice. Transcriptome sequencing revealed that the expression of apoptosis-related genes (*Capn1*, *Capn2*, *Bax*, *Aifm1, Caspase 3, Map3k5, Itpr1* and *Fas*) was down-regulated in spermatocytes of cryptorchid *Trpv1*^*−/−*^ mice. Our results suggest that TRPV1 promotes the apoptosis of spermatocytes in cryptorchid mice by regulating the expression of apoptosis-related genes.

## Introduction

Spermatogenesis is a heat-sensitive process, and the testicular temperature in most mammals must be maintained at a range from 2 to 8 °C below the core body temperature [1, 2]. Anything that raises the temperature of the testes and scrotum, including exposure to elevated ambient temperatures in occupational settings [3], clothing coverage [4], body posture [5], and pathologies such as varicocele [6] and cryptorchidism [7], could lead to the interruption of spermatogenesis in mammals. Studies have found that higher temperature can lead to a variety of adverse consequences in the testes, such as increasing oxidative stress levels [8], promoting DNA breaks and cell apoptosis [9]. Spermatogenic cells are highly susceptible to heat stress damage due to their frequent division [10]. It has been reported that meiosis in spermatocytes requires a low temperature to occur, and that spermatocytes in pachytene and diplotene stages are more susceptible to temperature variations [11]. At elevated testicular temperatures, double-strand breaks (DSBs) increase significantly, leading to an increase in aberrant chromosome binding and the elimination of heat-damaged spermatocytes via activation of meiotic checkpoints in the form of apoptosis [12].

Cryptorchidism is a common male genitourinary abnormality in which the testes do not descend completely but remain in the abdominal cavity or inguinal canal [13], leading to persistent damage to the spermatogenic epithelium and clinical infertility in men [14–16]. After surgery, 80% of adult men with a history of bilateral cryptorchidism and 30% of men with a history of unilateral cryptorchidism exhibit abnormal sperm counts [17]. In addition, abnormal testicular development and dysregulation of growth factor expression in cryptorchid males may lead to infertility and testicular cancer [18]. It has been established that the primary cause of impaired spermatogenesis in cryptorchidism is the exposure of the testis to the abdominal cavity, resulting in elevated temperatures. The surgery-induced cryptorchidism animal model induces an increase in scrotal temperature, which leads to the formation of multinucleated giant cells and the death of spermatogenic cells [19], ultimately causing a decrease in sperm production [20]. Apoptosis is generally considered the predominant mechanism of germ cell death in cryptorchidism, rather than atrophy or necrosis [21]. Furthermore, spermatogenic cell apoptosis is influenced by oxidative stress [22, 23], varying reactions to gonadotropins [24], and activation of nuclear factor κB in testes of experimentally induced cryptorchidism [25]. The primary apoptosis pathways of cryptorchid spermatogenic cells include the intrinsic mitochondrial pathway [26, 27], the Fas receptor system [28], and the p53 signaling pathway [26].

The heat-activated protein TRPV1, belonging to the TRP family, functions as a tetrametric transmembrane ion channel that remains closed and polarized at lower temperatures. The activation of TRPV1 is known to contribute significantly to the transduction of inflammatory pain [29–31]. Furthermore, it plays a crucial role in various processes, including apoptosis and cell proliferation [32, 33]. Additionally, it is involved in physiological functions such as thermoregulation [34]. In general, TRPV1 can be activated at temperatures greater than 42 °C [35]. Inflammatory conditions lower the temperature threshold for TRPV1 activation, resulting in its activation at body temperature [36]. In addition, it was found that TRPV1 channels can be rapidly activated at 33–39 °C in the presence of 2.5 μM phosphatidylinositol 4,5-bisphosphate [37]. *Trpv1*^−/−^ mice exhibit decreased sensitivity to heat and inflammation stimuli [38]. Due to its thermal sensitivity, TRPV1 has attracted increasing interest in the field of reproduction. Previous studies have demonstrated the predominant localization of TRPV1 in the cytoplasm of human testicular germ cells, the mid-posterior region of the sperm acrosome, and the flagella [39–41]. Furthermore, TRPV1 has been detected in the seminiferous epithelium and post-acrosomal region of the sperm in adult mice [42, 43]. Previous research showed that aged *Trpv1*^−/−^ mice exhibit a reproductive phenotype similar to that of youthful counterparts, characterized by reduced cell death in testicular spermatogenic cells [44]. Besides, the apoptosis of spermatogenic cells in *Trpv1*^*−/−*^ mice increased under heat stimulation at 42 °C, suggesting that TRPV1 plays a crucial role in the defense of testis against heat stress [45]. However, the TRPV1 agonist capsaicin can prompt the apoptosis of rat spermatogonium stem cells in vitro [46]. Together, these studies indicated that TRPV1 has different effects on spermatogenic cells under different stimulus conditions.

In this study, we determined that TRPV1 is predominantly expressed in the spermatocytes of the mouse testis and plays an important role in spermatogenic injury caused by cryptorchidism. We found that TRPV1 can promote spermatocyte apoptosis in cryptorchid mice by regulating the transcription of apoptosis-related genes. The study provides a new research perspective on the role of TRPV1 in male reproduction.

## Results

### TRPV1 is predominantly expressed in spermatocytes of mouse testis

**Fig. 1:**
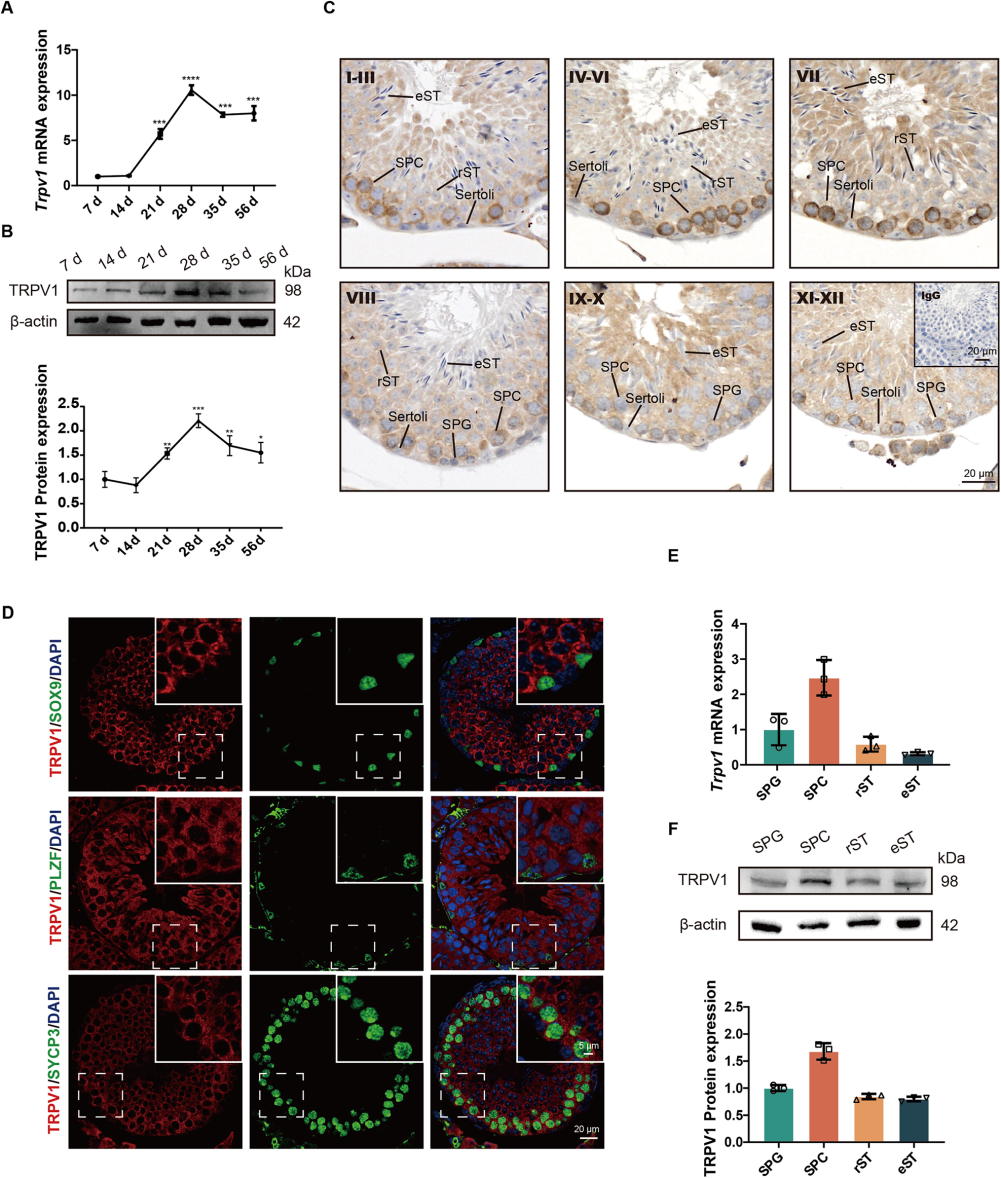
TRPV1 is mainly expressed on the cell membrane of mouse spermatocytes. **A**, **B** TRPV1 mRNA and protein expression along testicular development. **A** mRNA expression was assessed using RT-qPCR (*n* = 3). Amplification of *β-actin* mRNA was used as an internal control. **B** Protein expression was determined using Western blot (*n* = 3). Protein expression levels were normalized to β-actin and densitometric analyses were performed with ImageJ software. **C** Localization of TRPV1 protein in testicular sections from adult mice (8-week-old) was assessed using IHC assay. IgG is shown in the right lower panel. SPG spermatogonium, SPC spermatocyte, rST round spermatid, eST elongated spermatid. **D** Co-stained of TRPV1 (red) with Sertoli cell marker SOX9 (green), spermatogonium marker PLZF (green), and spermatocyte marker SYCP3 (green) in testicular sections of WT mice (8-week-old) was detected by IF. Nuclei were stained with DAPI (blue). Dashed boxes showed localization of the enlarged images. **E**, **F** The expression profile of TRPV1 in spermatogenic cells. Different types of spermatogenic cells in WT testes (8-week-old) were isolated using the STA-PUT method. E, mRNA expression was assessed using RT-qPCR (*n* = 3). Amplification of *β-actin* mRNA was used as an internal control. F, Protein expression was assessed using Immunoblot analysis (*n* = 3). Protein expression levels were normalized to β-actin and densitometric analyses were performed with ImageJ software. Data were represented as mean ± SD from three independent experiments. *p < 0.05, **p < 0.01, ***p < 0.001, ****p < 0.0001 by Student’s *t* test.

Previous studies have indicated that TRPV1 is expressed in the testicular tissues of humans [47] and mice [43], but the specific expression and localization of TRPV1 in germ cells of mice remain unclear. Therefore, we first investigated the expression and localization of TRPV1 in the mouse testis. RT-qPCR and Western blot analysis were performed using testicular tissues from 7-, 14-, 21-, 28-, 35-, and 56-day-old mice. The results showed that both TRPV1 mRNA and protein were expressed during testicular development in mice. Specifically, TRPV1 could be detected in the testis as early as postnatal day 7, gradually increased after day 14, reached a peak at day 28, and slightly decreased with the development of mice to adulthood (Fig. 1A, B). IHC analysis of TRPV1 in adult mice testis showed that the immunostaining signal of TRPV1 was observed in the membrane of spermatogenic cells at stages I to XII of the spermatogenic cycle, in which spermatocyte immunostaining was stronger at stages IV to VII and relatively weak at other stages (Fig. 1C). Co-staining of TRPV1 with SOX9 (Sertoli cell marker), PLZF (spermatogonium marker) and SYCP3 (spermatocyte marker) in testis of adult mice showed that TRPV1 was localized on the membranes of spermatogonia and spermatocytes, but not on Sertoli cells (Fig. 1D). In order to further understand the expression of TRPV1 in different spermatogenic cells, we isolated different types of spermatogenic cells, including spermatogonium (SPG), spermatocyte (SPC), round spermatid (rST) and elongated spermatid (eST) from adult mice testes. By RT-qPCR and Western blot analysis in these cells, we observed that TRPV1 was strongly expressed in spermatocytes and weakly expressed in other spermatogenic cells (Fig. 1E, F).

### Germ cell apoptosis is induced by experimental cryptorchidism

**Fig. 2:**
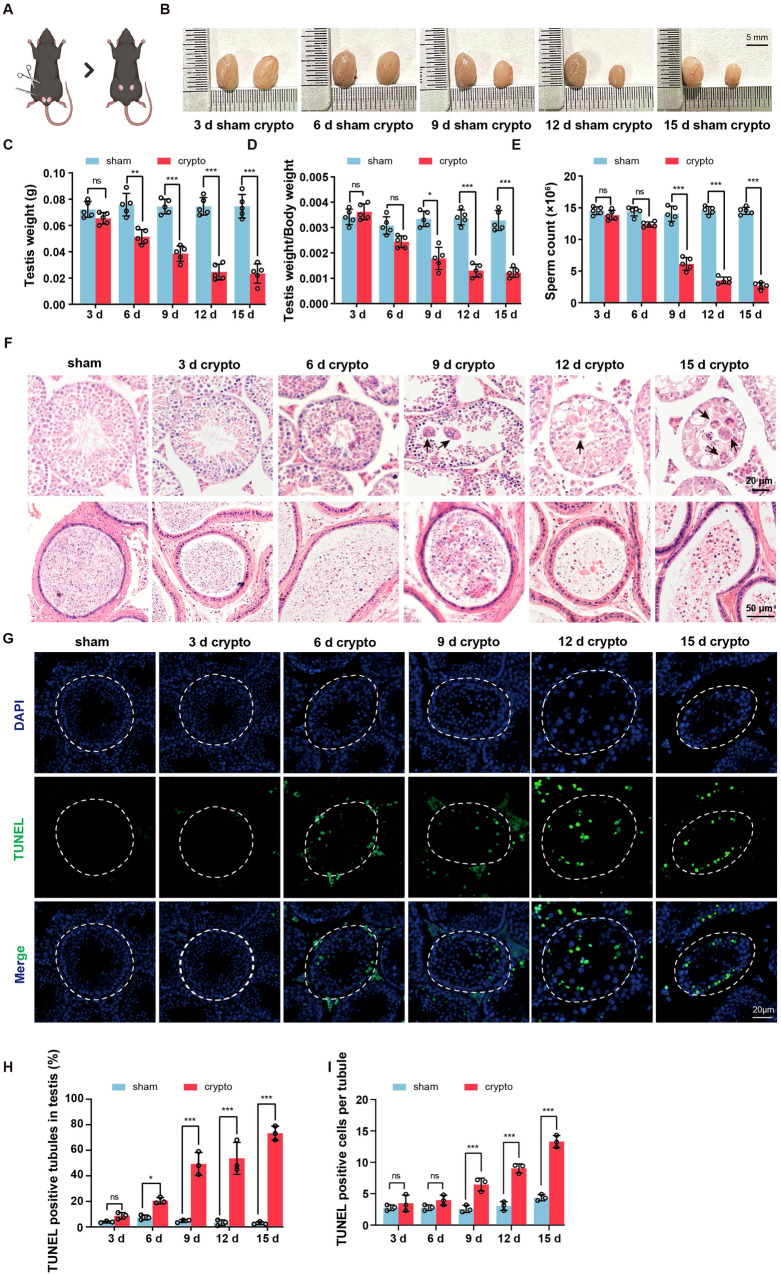
Apoptosis of spermatogenic cells increases with time in wild-type (WT) mice after cryptorchidism. **A** Schematic diagram of surgery-induced cryptorchidism in mice. Bilateral cryptorchidism was performed in 8-week-old mice. Comparison of the testicular size (**B**), testicular weight (**C**), testicular weight/body weight ratio (**D**) and the number of spermatozoa in cauda epididymis (**E**) on days 3, 6, 9, 12 and 15 after cryptorchidism (*n* = 5). **F** H&E staining of mouse testes and cauda epididymis on days 3, 6, 9, 12 and 15 after cryptorchidism. The arrows represent multinucleated giant cells. **G** Sections of testes on days 3, 6, 9, 12 and 15 after cryptorchidism were stained with the TUNEL probe (green), and nuclei were stained with DAPI (blue). Histogram showing the quantification of TUNEL positive tubules (**H**) and TUNEL positive cells per tubule (**I**) on days 3, 6, 9, 12 and 15 in cryptorchid testes (*n* = 3). Data were represented as mean ± SD from three independent experiments. *p < 0.05, **p < 0.01, ***p < 0.001 by Student’s *t* test.

Given the high sensitivity of testis to temperature, we hypothesized that TRPV1 may play a role in the apoptosis of testicular spermatogenic cells under heat stress. In the study, we established an endogenous heat injury apoptosis model using mouse experimental cryptorchidism. Bilateral cryptorchidism was surgically induced in 8-week-old mice (Fig. 2A), and the testicular volume, weight, the ratio of testis weight/body weight, and sperm count were measured on 3 days, 6 days, 9 days, 12 days, and 15 days after surgery. In cryptorchid mice, we observed a marked decline in testes volume from day 9 (Fig. 2B), testis weight began to decrease from day 6 and significantly reduced from day 9 until day 15 (Fig. 2C). Furthermore, the ratio of testis weight/body weight (Fig. 2D) and sperm count (Fig. 2E) declined from day 9 and continued to decline over time compared to the sham group. We then examined the morphology of the seminiferous tubules and cauda epididymis in mice after cryptorchidism. The H&E staining results showed that there were no significant differences in the morphology of seminiferous tubules and sperm count between the cryptorchidism group and the sham group on day 3. After 6 days of cryptorchidism surgery, spermatogenic cells began to degenerate, and immature and degenerative spermatogenic cell debris appeared in the lumen of epididymides. After 9 days of cryptorchidism, the seminiferous tubule further atrophied, and the number of spermatogenic cells in the seminiferous tubules decreased, while the number of multinucleated giant cells increased significantly. Additionally, immature and degenerative spermatogenic cell debris appeared in the lumen of epididymides. On day 12 and 15, there were only a few Sertoli cells and spermatogonia in the spermatogenic epithelium of cryptorchid mouse, and the amount of spermatogenic cell debris in the epididymal lumen increased further (Fig. 2F). In order to observe the apoptosis of spermatogenic cells more directly, we used TUNEL to analyze the seminiferous tubules of cryptorchid testis. The results showed that spermatogenic cells began apoptosis on the 6 days after cryptorchidism, and the number of apoptosis cells increased with the duration of cryptorchidism. Both the number of TUNEL positive cells per tubule and the number of TUNEL positive tubules were significantly increased in the day 9 cryptorchid testis compared with the sham group (Fig. 2G–I). Altogether, these findings indicate that serious damage to spermatogenic cells begins at day 9 after cryptorchid surgery.

### TRPV1 is upregulated in cryptorchid testis

**Fig. 3:**
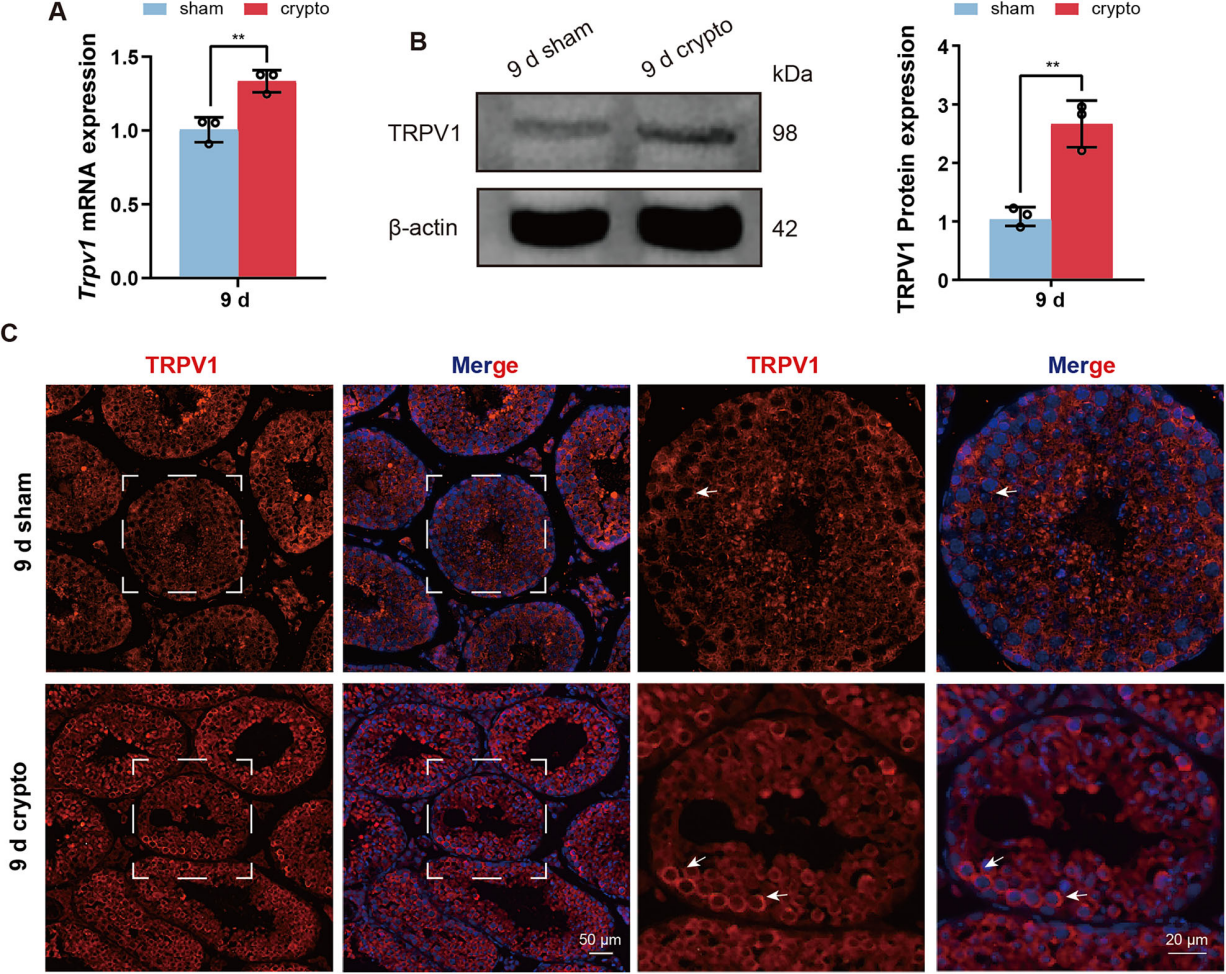
TRPV1 expression increases in the testis on day 9 after cryptorchidism. **A**, **B** TRPV1 mRNA and protein expression in the testis on day 9 after cryptorchidism (*n* = 3). **A** mRNA expression was assessed using RT-qPCR. Amplification of *β-actin* mRNA was used as an internal control. **B** Protein expression was determined using Western blot. Protein expression levels were normalized to β-actin and densitometric analyses were performed with ImageJ software. **C** IF staining was applied to detect TRPV1 (red) in the 9-day cryptorchid testis. Dashed boxes showed localization of the enlarged images. White arrows indicated cells with strong positive signals. Data were represented as mean ± SD from three independent experiments. **p < 0.01 by Student’s *t* test.

To investigate whether TRPV1 is related to the apoptosis of spermatogenic cells induced by cryptorchidism, the expression profile of TRPV1 in testes was detected on days 9 after cryptorchidism. RT-qPCR and Western blot analysis indicated that TRPV1 mRNA and protein expression levels were moderate in the testes of sham group, but significantly up-regulated in the cryptorchid testes (Fig. 3A, B). IF staining also revealed increased TRPV1 fluorescence intensity at 9 days after cryptorchid surgery compared to the sham group (Fig. 3C).

### TRPV1 is dispensable for male fertility

**Fig. 4:**
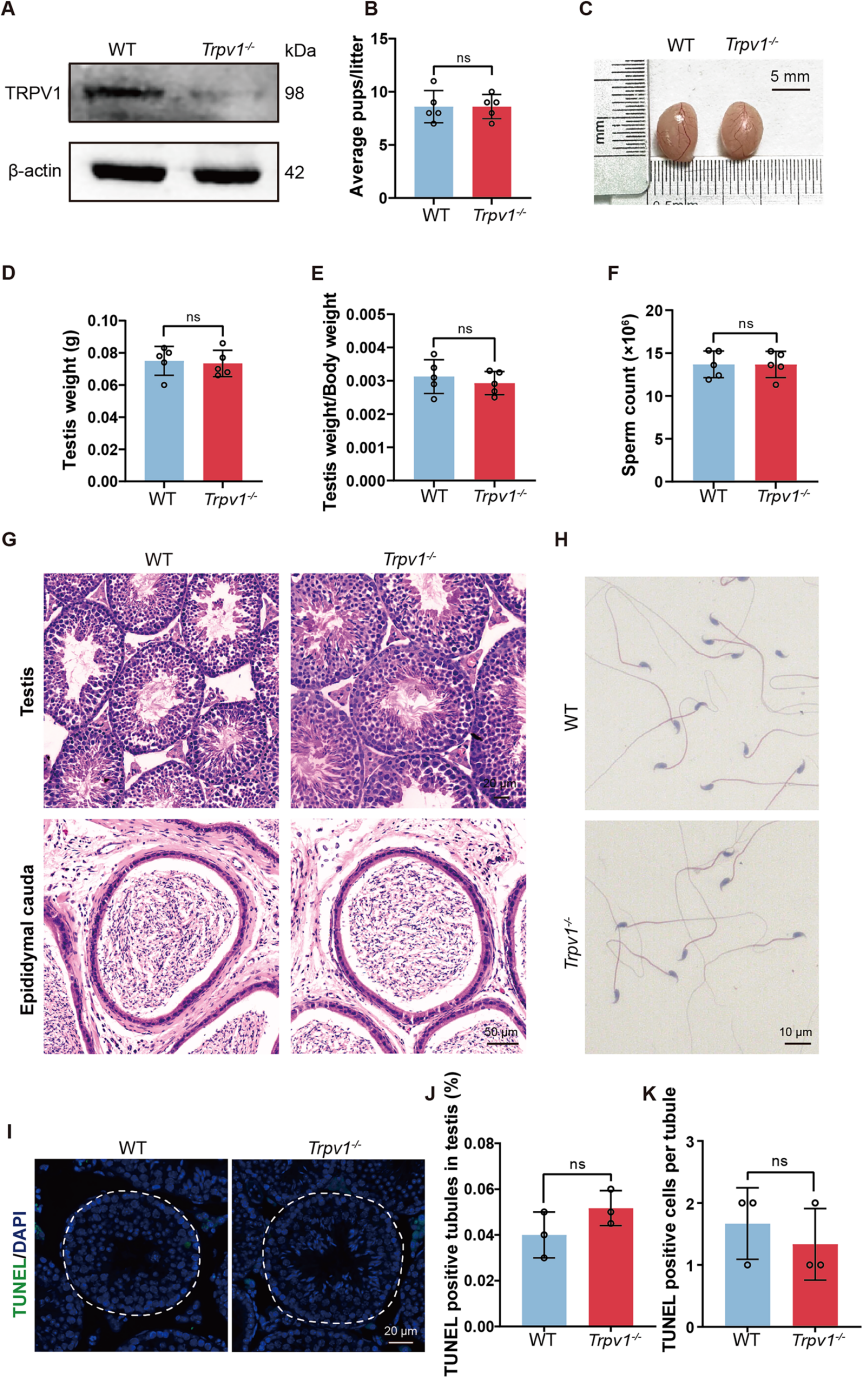
Knockout of *Trpv1* does not affect sperm development and mice fertility. **A** Protein expression of TRPV1 in the testes of WT and *Trpv1*^*−/−*^ mice was determined using Western blot. β-actin served as a loading control. Comparison of average pups (**B**), testicular size (**C**), testicular weight (**D**), testicular weight/body weight ratio (**E**), and the sperm count in cauda epididymis (**F**) of 8-week-old *Trpv1*^*−/−*^ and WT mice (*n* = 5). **G** H&E staining of testis and cauda epididymis of 8-week-old *Trpv1*^*−/−*^ and WT mice. **H** H&E staining of spermatozoa from 8-week-old *Trpv1*^*−/−*^ and WT mice. **I** Sections of testes from 8-week-old WT mice and *Trpv1*^*−/−*^ mice were stained with a TUNEL probe (green), and nuclei were stained with DAPI (blue). Histogram showing the quantification of TUNEL positive tubules (**J**) and TUNEL positive cells per tubule (**K**) of 8-week-old WT mice and *Trpv1*^*−/−*^ mice (*n* = 3). Data were represented as mean ± SD from three independent experiments. The data were analyzed with Student’s *t* test. ns not significant.

To further study the relationship between TRPV1 and spermatogenic cell apoptosis, we obtained the *Trpv1*^*+/−*^ mice from the Jackson laboratory. *Trpv1*^*+/−*^ male mice were bred with *Trpv1*^*+/−*^ female mice to generate *Trpv1*^*−/−*^ mice. We first used Western blot to confirm the knockout efficiency of TRPV1 in the *Trpv1*^*−/−*^ testis. The result showed that TRPV1 was expressed in wild-type (WT) mice testis, but almost disappeared in *Trpv1*^*−/−*^ mice testis (Fig. 4A). Then, we explored the reproductive phenotypes of *Trpv1*^*−/−*^ mice. We found that there were no differences in the litter size between *Trpv1*^*−/−*^ mice and WT mice (Fig. 4B). Besides, the testicular volume, testicular weight, testicular weight/body weight ratio and sperm counts of *Trpv1*^*−/−*^ mice were comparable to the WT mice (Fig. 4C–F). Furthermore, the histological analysis of *Trpv1*^*−/−*^ mice also did not reveal any remarkable abnormalities in the testis, cauda epididymis and sperm of *Trpv1*^*−/−*^ mice (Fig. 4G, H). Moreover, we found that apoptosis in spermatogenic cells of *Trpv1*^*−/−*^ mice and WT mice had no significant difference (Fig. 4I–K). Hence, we conclude that TRPV1 is dispensable for male fertility in normal conditions.

### The deficiency of TRPV1 alleviates reproductive damage induced by experimental cryptorchidism

**Fig. 5:**
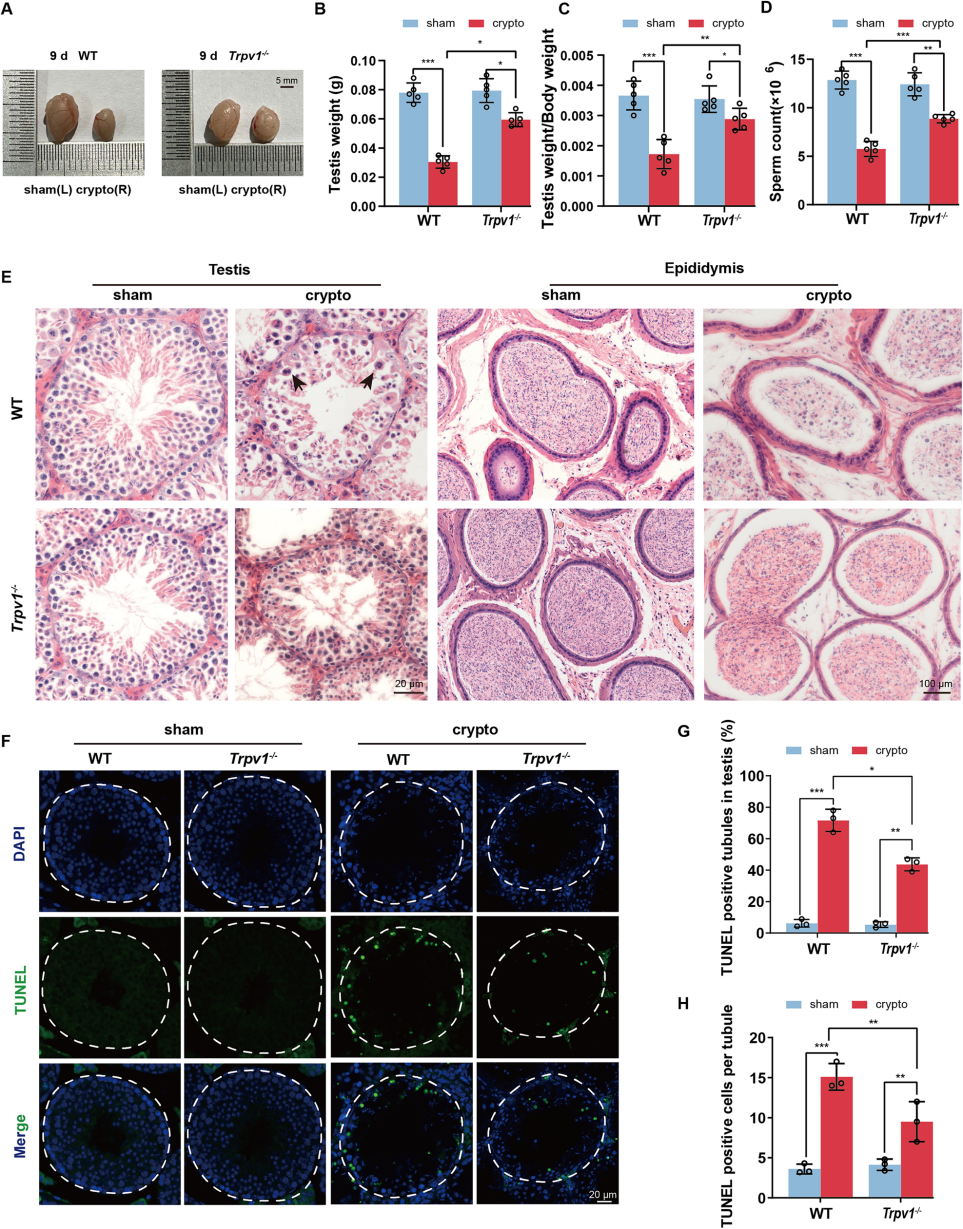
The apoptosis of spermatocytes decreases after 9 days of cryptorchidism in *Trpv1*^*−/−*^ mice. Comparison of the testicular size (**A**), testicular weight (**B**), testicular weight/body weight ratio (**C**), and the number of spermatozoa in cauda epididymis (**D**) on day 9 after cryptorchidism in 8-week-old WT mice and *Trpv1*^*−/−*^ mice (*n* = 5). **E** H&E staining of testes and cauda epididymis in cryptorchid surgery groups and sham groups of 8-week-old WT mice and *Trpv1*^*−/−*^ mice. **F** Sections of testes from 8-week-old WT mice and *Trpv1*^*−/−*^ mice on day 9 after cryptorchidism were stained with a TUNEL probe (green), and nuclei were stained with DAPI (blue). Histogram showing the quantification of TUNEL positive tubules (**G**) and TUNEL positive cells per tubule (**H**) on day 9 of cryptorchid testis in 8-week-old WT mice and *Trpv1*^*−/−*^ mice (*n* = 3). Data were represented as mean ± SD from three independent experiments. *p < 0.05, **p < 0.01, ***p < 0.001 by Student’s *t* test.

To reveal the function of TRPV1 in spermatogenic cell apoptosis after cryptorchidism, we further compared the reproductive phenotypes of *Trpv1*^*−/−*^ mice and WT mice at 9 days after cryptorchidism. We found that the testicular volume of *Trpv1*^*−/−*^ cryptorchid mice decreased less than that of WT cryptorchid mice (Fig. 5A). In addition, the testis weight, the ratio of testis weight/body weight and sperm count were increased in *Trpv1*^*−/−*^ cryptorchid mice compared with WT cryptorchid mice, suggesting that the injury of cryptorchid testis was reduced after TRPV1 deletion (Fig. 5B–D). H&E staining showed that the seminiferous tubules had fewer luminal vacuoles and multinucleated giant cells in *Trpv1*^*−/−*^ mice compared with WT mice after cryptorchidism. Besides, compared with WT cryptorchid mice, *Trpv1*^*−/−*^ cryptorchid mice had more spermatogenic cells in the testis and more sperm in cauda epididymis (Fig. 5E). Next, we performed TUNEL staining of testes from *Trpv1*^*−/−*^ mice and WT mice after cryptorchid surgery. Our results showed that positive TUNEL signals were reduced in *Trpv1*^*−/−*^ cryptorchid mice (Fig. 5F). Statistical analysis of TUNEL staining showed that the number of TUNEL positive cells (Fig. 5G) and positive tubules (Fig. 5H) were significantly reduced in *Trpv1*^*−/−*^ mice compared to WT mice after cryptorchid surgery. We further observed by periodic acid-schiff (PAS)-hematoxylin and PNA staining that compared with WT cryptorchid mice, the stages of seminiferous epithelium in *Trpv1*^*−/−*^ mice after cryptorchid were recovered, and the acrosome of sperm was well developed (Supplementary Fig. S1A, B). These findings suggest that TRPV1 plays a role in promoting the apoptosis of spermatogenic cells after cryptorchid surgery.

### Apoptosis-related genes are downregulated in the spermatocytes of *Trpv1*^−/−^ mice with cryptorchidism

**Fig. 6:**
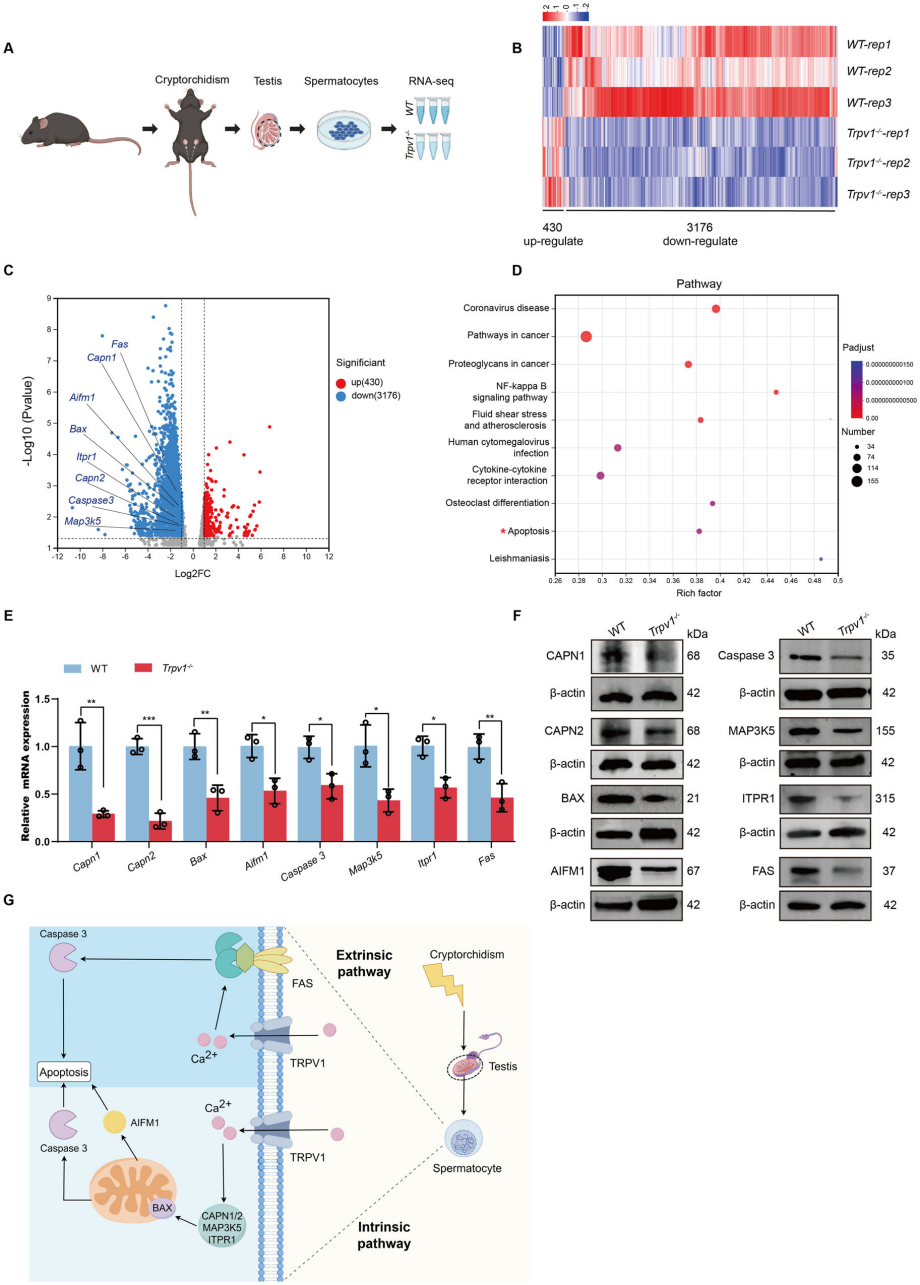
*Trpv1*^*−/−*^ mice exhibit downregulated expression of apoptosis-related genes in spermatocytes after cryptorchidism. **A** Schematic diagram depicting transcriptome analysis of spermatocytes. Briefly, total RNA from WT and *Trpv1*^*−/−*^ spermatocytes of cryptorchid testes on day 9 were collected for the RNA-seq. **B** Heatmaps depicting significantly upregulated and downregulated genes in WT and *Trpv1*^*−/−*^ spermatocytes. The genes with |log2 Fold change|≥1 and *q* < 0.05 were determined to generate the heatmap. **C** Volcano plot showing DEGs in WT and *Trpv1*^*−/−*^ spermatocytes. The significant changed downregulated genes associated with apoptosis were labeled with gene name. **D** KEGG pathway analysis of 3176 downregulated genes in *Trpv1*^*−/−*^ spermatocytes. The apoptosis pathway was labeled with asterisks. **E**, **F** Expression of CAPN1, CAPN2, BAX, AIFM1, Caspase 3, MAP3K5, ITPR1 and FAS in spermatocytes of WT and *Trpv1*^*−/−*^ cryptorchid mice were detected by RT-qPCR and Western blot analysis (*n* = 3). Data were represented as mean ± SD from three independent experiments. *p < 0.05, **p < 0.01, ***p < 0.001 by Student’s *t* test. **G** Cryptorchidism induces the increase of TRPV1 expression in mouse spermatocytes, leading to the increase of [Ca^2+^]_i_, which participates in the endogenous mitochondrial apoptosis pathway through up-regulation of CAPN1, CAPN2, BAX, AIFM1, MAP3K5 and ITPR1 on the one hand, and in exogenous apoptosis pathway through up-regulation of FAS on the other hand, ultimately leading to spermatocyte apoptosis. This picture was drawn by figdraw.

To gain insight into the molecular mechanism underlying TRPV1 regulation of spermatocyte apoptosis in cryptorchid mice, we used purified spermatocytes obtained from the testes of *Trpv1*^*−/−*^ and WT mice at 9 days after cryptorchidism and extracted total RNA for RNA-seq (Fig. 6A). Transcriptome analysis showed that 430 genes were up-regulated and 3176 genes were down-regulated in spermatocytes of *Trpv1*^*−/−*^ cryptorchid mice compared with WT mice (Fig. 6B, C). KEGG pathway analysis revealed that the apoptosis pathway was enriched in the down-regulated DEGs, and the major down-regulated genes in apoptosis pathways included *Capn1*, *Capn2*, *Bax*, *Aifm1, Caspase 3, Map3k5, Itpr1* and *Fas* (Fig. 6C, D). To validate the RNA-seq findings, we conducted RT-qPCR and Western blot analyses on these genes. The results confirmed that the mRNA and protein levels of these genes were significantly decreased in spermatocytes of *Trpv1*^*−/−*^ cryptorchid mice (Fig. 6E, F), which was consistent with the RNA-seq results. Hence, we conclude that TRPV1 can induce the increased expression of apoptosis-related molecules in the mouse model of cryptorchidism to participate in spermatocyte apoptosis.

## Discussion

Cryptorchidism is a common congenital disease of the male reproductive system, in which the testes are exposed to a relatively high-temperature environment, leading to apoptosis of spermatogenic cells and subsequent infertility. However, the underlying mechanism of spermatogenic cell apoptosis caused by increased testicular temperature remains unclear. In this study, we observed the phenotype of *Trpv1*^*−/−*^ cryptorchid mice and found that TRPV1 knockout can alleviate the reduction in sperm count, damage to the seminiferous epithelium, and spermatogenic apoptosis caused by cryptorchidism. Furthermore, we preliminarily elucidated the molecular mechanism by which TRPV1 exerts pro-apoptotic effects on spermatogenic cells in response to thermal stimulation by regulating apoptotic molecules in both the death receptor pathway and mitochondrial pathway of apoptosis.

In this study, we observed that TRPV1 expression was detected in the testes of mice at day 7, significantly increased from day 21 to day 28, and then gradually decreased as the mice became adults. Throughout the developmental stages of mice, all types of spermatogonia (undifferentiated, A1, A2, A3, A4, intermediate, and B spermatogonia) were seen in the testis as early as postnatal day 5–6, followed by the differentiation of spermatogonia and meiosis in spermatocytes, and then by day 27, the first elongated spermatids were observed [48]. The expression profile of TRPV1 during postnatal testicular development suggests its potential significance in spermatogenesis. Our further examination showed that TRPV1 was expressed on the cell membrane of all spermatogenic cells, and the expression was relatively high in primary spermatocytes, suggesting that TRPV1 may play an important role in spermatocytes. Nevertheless, we found that the male *Trpv1*^*−/−*^ mice exhibited normal spermatogenesis and fertility. The number of litters, testis weight, testis weight/body weight, and sperm count of *Trpv1*^*−/−*^ mice were not different from those of WT mice. Moreover, no significant morphological abnormality was observed in the testis, epididymis, and sperm in *Trpv1*^*−/−*^ mice. These findings suggest that knocking out TRPV1 does not affect mouse fertility, which is consistent with a previous report [38].

Thermal stimulation is considered a significant factor leading to reproductive damage following cryptorchidism. Moreover, spermatogenic cells are more susceptible to apoptosis induced by thermal stimulation than other cells, attributable to their high rate of division [49]. TRPV1, as a thermoreceptor, plays an important role in sensing temperature [50]. Therefore, we examined the function of TRPV1 in cryptorchid mice. We found that cryptorchid mice at 9 days had significantly decreased testicular volume, testis weight/body weight, and sperm count in cauda epididymis, with a large amount of spermatogenic epithelium detached, multinucleated giant cells in the lumen of the seminiferous tubules, and a significant increase in the number of apoptotic spermatogenic cells. The presence of multinucleated giant cells and apoptosis in spermatogenic cells are indicative morphological features of seminiferous epithelium damage in cryptorchid testes [19]. Our results show that severe damage to spermatogenic cells begins at 9 days of cryptorchidism. Moreover, our investigation of TRPV1 expression at 9 days of cryptorchidism revealed a significant increase in its levels within cryptorchid testes, which suggests that TRPV1 may be involved in spermatogenic cell damage caused by cryptorchidism. In order to clarify the role of TRPV1 in spermatogenic damage caused by cryptorchidism, we further constructed the cryptorchid model of *Trpv1*^*−/−*^ mice, and found that there were obvious phenotypic differences between *Trpv1*^*−/−*^ mice and WT mice after cryptorchidism. In *Trpv1*^*−/−*^ cryptorchid mice, the damage of the seminiferous epithelium, the reduction of spermatozoa in cauda epididymis and the apoptosis of spermatogenic cells were all less severe than those in WT cryptorchid mice. It is evident that TRPV1 promotes spermatogenic cell apoptosis in cryptorchid mice, which is consistent with the results that TRPV1 promotes testicular cell apoptosis in aged mice found by Adrian S. Siregar et al. [44]. However, it is worth noting that the role of TRPV1 on spermatogenic apoptosis under different conditions appears to be quite complex. When the scrotum of *Trpv1*^*−/−*^ mice was exposed to a 42 °C hot water bath, spermatogenic apoptosis increased, indicating that TRPV1 inhibits spermatogenic apoptosis under 42 °C thermal stimulation [45], which was contrary to our findings that TRPV1 could promote spermatogenic apoptosis under cryptorchidism. This difference suggests that the effect of TRPV1 on spermatogenic apoptosis depends on the specific activation conditions.

Cryptorchidism induces germ cell apoptosis, which mainly affects spermatocytes and round spermatids, eventually leading to testicular atrophy [51, 52]. Given our observation of high TRPV1 expression in spermatocytes, we further investigated the molecular mechanisms underlying TRPV1-induced spermatocyte apoptosis in cryptorchidism. TRPV1 is a ligand-gated cation channel, whose activation results in Ca^2+^ influx into the cell [53]. Ca^2+^ signals are involved in important checkpoints in cell death pathways and promote apoptosis [54]. There are two main pathways of apoptosis, intrinsic mitochondria-mediated pathway and extrinsic death receptor-mediated pathway [55]. Our RNA-Seq data revealed that genes related to apoptosis, such as *Capn1*, *Capn2*, *Bax*, *Aifm1, Caspase 3, Map3k5, Itpr1* and *Fas* were reduced in the spermatocytes of *Trpv1*^*−/−*^ cryptorchid mice. CAPN1 and CAPN2 are Ca^2+^ dependent cysteine proteases that cleave and activate Bax, allowing the translocation of Bax to the mitochondria [56, 57]. Bax is a pro-apoptotic protein, primarily functioning to enhance the permeability of the mitochondrial membrane [58]. The damage to the mitochondria leads to the exposure of their inner membrane, which causes the release of mitochondrial intermembrane space proteins that can either trigger caspase 3 activation, or caspase-independent pathways, such as AIFM1 [59]. Previous study has shown that Ca²⁺ influx induced by TRPV1 causes mitochondrial hyperpolarization and depolarization, leading to upregulation of Bax and release of AIFM1 [53]. This is consistent with our finding that the expression of Bax and AIFM1 is reduced in spermatocytes of *Trpv1*^*−/−*^ cryptorchid mice. MAP3K5, known as apoptosis signal-regulating kinase 1, can also be activated in response to calcium overload [60], leading to mitochondrial dysregulation and eventually apoptosis [61]. ITPR1 is an intracellular Ca^2+^ release channel that regulates mitochondrial calcium-dependent apoptosis by facilitating calcium transport from the endoplasmic reticulum lumen to the mitochondria intermembrane space [62]. One study has found that the elevated apoptosis of spermatogenic cells in non-obstructive azoospermia patients was associated with increased expression of ITPR1 and Bax [63]. Fas functions as a death receptor in the extrinsic pathway of apoptosis. Upon binding to its ligand FASL, Fas initiates the caspase cascade to drive apoptosis [64]. In this study, reduced expression of Fas was detected in the spermatocytes of *Trpv1*^*−/−*^ cryptorchid mice. This corresponds to a previous study that [Ca^2+^]_i_ induced by TRPV1 activation could promote the aggregation of Fas death receptors and induce apoptosis in urothelial cancer cells [65]. Threrfore, our results suggest that TRPV1 is involved in spermatocyte apoptosis in mice with cryptorchidism through both intrinsic mitochondrial-mediated pathway and extrinsic death receptor-mediated pathway (Fig. 6G). Further molecular mechanisms need to be explored in future studies.

In summary, our study clarifies that the thermosensitive protein TRPV1 is mainly expressed on the membrane of mouse spermatocytes. And under cryptorchid conditions, TRPV1 can enhance the transcription of apoptotic molecules through both mitochondrial and death receptor apoptosis pathways, leading to spermatogenic cell apoptosis. This study demonstrates for the first time that TRPV1 plays an important role in spermatogenic cell apoptosis after cryptorchidism, providing a new target for the treatment of cryptorchidism.

## Materials and methods

### Animals

WT (C57BL/6J) mice were obtained from the Laboratory Animal Center of Air Force Military Medical University, and *Trpv1*^*+/−*^ mice (Strain#: 003770) were obtained from Jackson Laboratory. All mice were housed in a specific pathogen-free (SPF) environments with a 12 h:12 h light/dark cycle and an ambient temperature of 23 ± 2 °C. Mice were randomly assigned to different experimental groups.

### Reverse transcriptase - quantitative PCR (RT-qPCR)

Total RNA was extracted from the testes and spermatogenic cells using Trizol (Takara, Kyoto, Japan). RNA was reverse transcribed into cDNA using SmArt RT Master Premix (5×) (Deeyee, Shanghai, China) according to the manufacturer’s instructions. The synthesized cDNA was amplified with specific primers (Supplementary Table S1) and SYBR Green (Deeyee, Shanghai, China) using a QuantStudio 3 real-time fluorescence quantitative PCR system (Thermo Fisher Scientific, MA, USA). Triplicate samples were examined for each condition. The relative mRNA expression level was calculated using the 2^−ΔΔCt^ method.

### Western blot

Total proteins were extracted from testes or spermatogenic cells using cold-RIPA buffer (Beytime, Shanghai, China) with 1 mM PMSF (Beytime, Shanghai, China) and 1 mM protease inhibitor cocktail (Solarbio, Beijing, China). The lysates were centrifuged at 12,000 × *g* for 15 min. The protein concentration was determined using a BCA Protein Detection Kit (Beyotime, Shanghai, China). Proteins were separated by SDS-PAGE electrophoresis and transferred to a nitrocellulose membrane. The membrane was blocked with 5% (w/v) nonfat milk at room temperature for 1 h and then incubated with the appropriate primary antibodies at 4 °C overnight. Then, the membrane was incubated with HRP-conjugated secondary antibody at room temperature for 1 h. Protein bands were visualized using an enhanced chemiluminescence kit (InCellGenE, TX, USA) and the membrane was photographed by Clinx chemiluminescence instrument (Clinx Science Instruments Co., Ltd, Shanghai, China). The antibodies used in this study are listed in Table S2. Uncropped immunoblot gels are shown in supplementary file.

### Tissue fixation and histological analysis

Testes and caudal epididymides were fixed in 4% (m/v) paraformaldehyde (PFA) for 24 h, dehydrated with gradient alcohol, embedded in paraffin, and processed into 5 μm sections. After deparaffinization, the tissue sections were stained with hematoxylin and eosin (H&E). For periodic acid-Schiff (PAS) staining, deparaffinized slides were stained with PAS and hematoxylin according to the protocol of PAS kit (G1280, Solarbio, China).

### Immunofluorescence (IF), immunohistochemistry (IHC) and TUNEL assay

For IF assay, after routine deparaffin and rehydration, testicular sections were microwave-repaired in 10 mM sodium citrate solution (pH 6.0) for 15 min. Sections were block with 5% (m/v) BSA (Biofroxx, Einhausen, DE) for 30 min and incubated overnight at 4 °C with the appropriate primary antibodies. Subsequently, the sections were incubated with the secondary antibody at room temperature for 2 h. PNA dye (Sigma, MO, USA) was used to label the acrosomes of sperm cells at room temperature for 2 h. DAPI (abcam, Cambridge, UK) staining was used to visualize the nuclei of cells. The sections were mounted in 50% (v/v) glycerol and examined with a VS200 microscope (Olympus, Tokyo, Japan) and a FV1000 confocal microscope (Olympus, Tokyo, Japan).

For IHC assay, testicular sections were routinely deparaffinized, rehydrated, and microwave-repaired. Sections were incubated in 0.3% (m/v) H_2_O_2_ for 30 min at room temperature, and blocked with 5% (m/v) BSA at room temperature for 30 min. Sections were incubated with primary antibody at 4 °C overnight. Control sections were incubated with serum from the same source as the primary antibody instead of the primary antibody, followed by sequential incubation with biotinylated secondary antibody and streptavidin peroxidase complex provided with the VECTASTAIN Elite ABC HRP Kit (PK-4001, VECTASTAIN, USA). Sections were incubated with 0.02% (m/v) DAB (Sigma, MO, USA) solution, followed by staining of nuclei with hematoxylin for 1 min. And images were acquired using VS200 microscope (Olympus, Tokyo, Japan). The antibodies used for IF and IHC assay in this study are listed in Table S2.

For TUNEL assay, after the routinely deparaffinized, rehydrated, and microwave-repaired, enzyme and labeling solutions were mixed followed the TUNEL kit protocol (Roche, Basel, CH). Then, testicular sections were incubated in the mixture for 1.5 h at 37 °C under dark conditions. DAPI was used to stain the nuclei. Images were acquired using a VS200 microscope (Olympus, Tokyo, Japan).

### Separation of spermatogenic cells

Spermatogenic cells were isolated by the STA-PUT method as previously described [66]. In brief, mouse testes were suspended in DMEM medium (Gibco, NYC, USA) containing 1.5 mg/ml hyaluronidase (InCellGenE, TX, USA) and 1.5 mg/ml collagenase IV (Gibco, NYC, USA). The sample was then digested in a 37 °C-water bath for 15 min. The digested cells were centrifuged and filtered through a 200-mesh filter (Solarbio, Beijing, China). A 2–4% (m/v) BSA gradient was applied to the separation tube, and the cells were left in the separation tube for 3 h. Different types of cells were collected from the bottom of the separator. The purity and type of cells in each tube were assessed by light microscopy according to the size and morphological characteristics of the cells. The collected cells were used for subsequent experiments.

### Surgery-induced cryptorchidism

Eight-week-old WT or *Trpv1*^−/−^ mice were randomly assigned to sham and cryptorchid groups. For the cryptorchid group, mice were anesthetized with 3% pentobarbital sodium. A cut was made along the skin in the right and left upper abdominal region and the adipose tissue of the caput epididymis was sutured to the inner peritoneal wall. For the sham group, mice were also anesthetized and bilateral abdominal incisions were made, but no further treatment was applied before suturing. The testes and epididymides of the mice were collected on days 3, 6, 9, 12, and 15 after cryptorchidism.

### Fertility assay

Male WT mice or *Trpv1*^*−/−*^ mice were mated with WT female mice at a ratio of 1:2. Vaginal plugs were checked the next day. After mating, each male mouse rested separately for 1–2 days before starting the next round of mating. The number of litters of female mice with positive vaginal plugs were recorded.

### Sperm collection, sperm count and morphology test

The cauda epididymis of WT mice or *Trpv1*^*−/−*^ mice was incised, and the spermatozoa were extruded in PBS. The spermatozoa were incubated for 10 min at 37 °C in a CO_2_ incubator, and then filtered through a 100 μm aperture cell sieve. Sperm counts were performed using a hemocytometer under a microscope. Additionally, sperm samples from the cauda epididymis of WT mice or *Trpv1*^*−/−*^ mice were smeared onto slides, fixed for 30 min with 4% (m/v) paraformaldehyde in PBS, then rinsed and subjected to H&E staining.

### Isolation of primary spermatocytes

Isolation of primary spermatocytes was performed as described as in a previous article [67]. In brief, mouse testes were isolated, digested with 1× Krebs buffer containing 1 mg/ml collagenase IV (InCellGenE, Texas, USA) at 37 °C for 3 min. Subsequently, 1× Krebs buffer was added to the sample and left for 1 min at room temperature. The sample was then centrifuged at 600 g for 5 min, and the supernatant was discarded. Afterward, the sample was diluted with 1× Krebs buffer containing 0.6 mg/ml trypsin (Solarbio, Beijing, China) and digested on a tube rotator inside the 34 °C incubator for 15–20 min. The sample was then filtered through a 40 μm cell strainer (Biosharp, Anhui, China). The sample was centrifuged again at 600 × *g* for 5 min, the supernatant was discarded, and this step was repeated 2–3 times. A gradient of 1–5% concentration of BSA was prepared, the cells were resuspended with 0.5% BSA, and loaded onto the gradient. After 1.5 h, the cells were collected and observed under a microscope.

### RNA-seq library construction and data analysis

Total RNA from spermatocytes of *Trpv1*^*−/−*^ and WT mice testes at 9 days after cryptorchidism was extracted using Trizol. Libraries were constructed using the TruseqTM RNA Library Preparation Kit (Illumina, CA, USA). Sequencing was performed on the Illumina Novaseq 6000. Data quality control was constructed by comparing the fast and pure data to the reference genome Mus_musculus. Gene expression was analyzed using RSEM. DEGseq was used to analyze differential gene expression between biological replicates. Genes with |log2 fold change|≥1 and *p* (value) <0.05 were identified as differentially expressed genes. The sequencing and analysis were performed by Megi Biomedicine Technology Co., Ltd (Shanghai, China).

### Statistical analysis

All quantitative analyses were performed under blinded conditions. Detailed *n* values for each panel in the figures were stated in the corresponding legends. Sample sizes were based in standard protocols in the field and no sample or animal was excluded. Quantitative data with normal distribution were expressed as mean ± SD and Student’s *t* test was used for comparison between groups. All statistical analyses were performed by GraphPad Prism 9.0 Software. *P* < 0.05 was considered statistically significant.

## Supplementary information

Supplementary figure and table

Original Western Blots

## Data Availability

All data generated or analyzed during this study are included in this published article and its supplementary information files. The RNA-seq datasets generated for this study can be found in the SRA metadata under accession PRJNA1216033.
