# Supplementary material for: The involvement of TRPV1 in the apoptosis of spermatogenic cells in the testis of mice with cryptorchidism
Source: Cell Death Discov. 2025 Apr 3;11:135. doi: 10.1038/s41420-025-02447-3 (PMC11968804; doi:10.1038/s41420-025-02447-3)
Supplement: Supplementary file 1 — Supplementary figure and table [file 41420_2025_2447_MOESM1_ESM.docx]

Supplementary Information

The involvement of TRPV1 in the apoptosis of spermatogenic cells in the testis of mice with cryptorchidism

Yanqiu Zhao^1,3^, Jinhua Wei^1,3^, Pang Cheng^1,3^, Junxian Ma^1^, Bo Liu^1,2^, Mingxiang Xiong^1^, Ting Gao^1^, Jingqi Yao^1^, Tianchen Sun^1^, Zhen Li^1*^

^1^ Department of Human Anatomy and Histo-embryology, Air Force Medical University, 710032 Xi’an, China

^2^ The Air Force Hospital of Central Theater of PLA, 037000 Datong, China

* Corresponding author: Zhen Li, Email: [lizhenhe@fmmu.edu.cn](mailto:lizhenhe@fmmu.edu.cn)

Yanqiu Zhao, Jinhua Wei, Pang Cheng contributed equally to this work and should be considered as equal first authors.


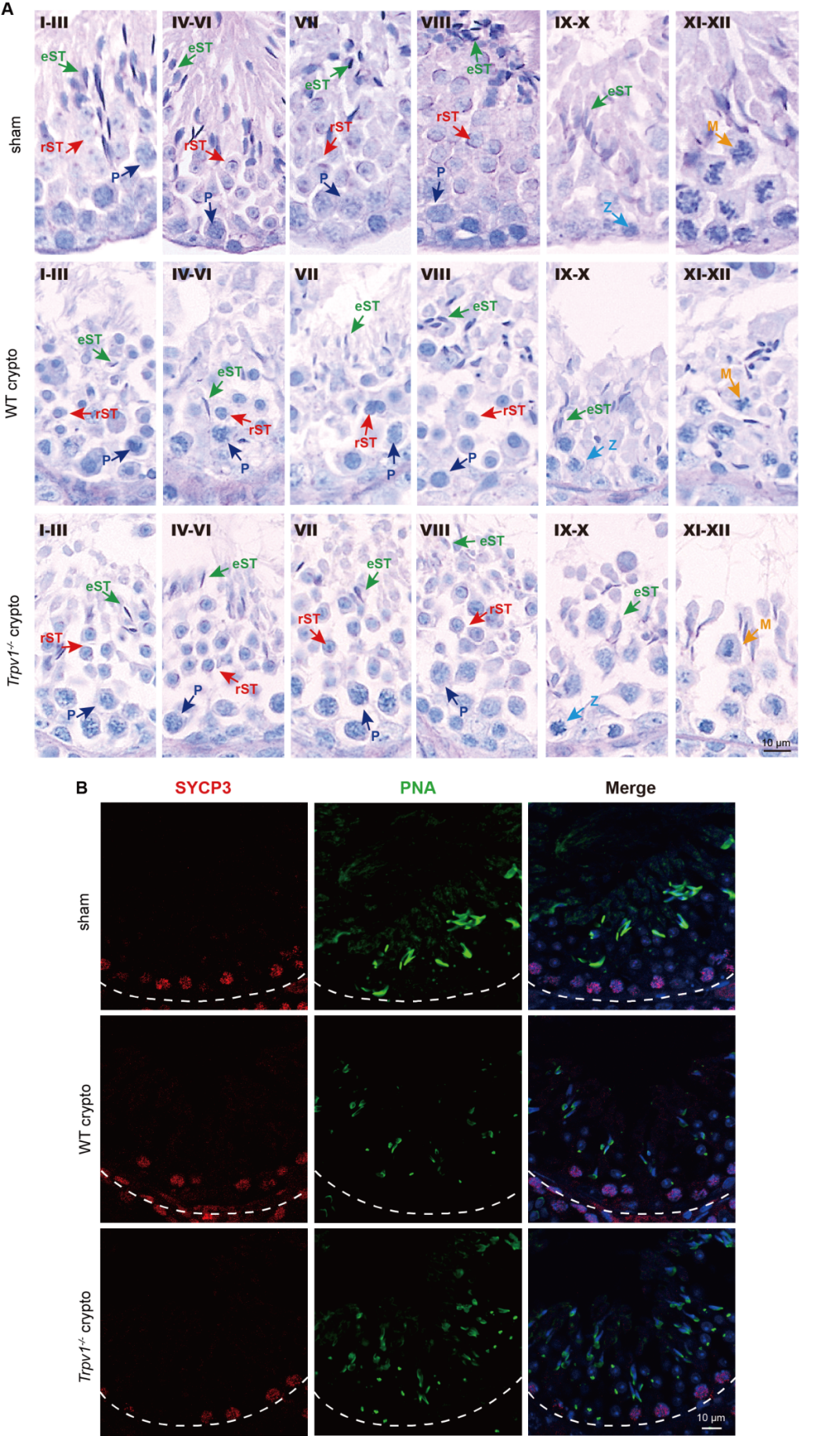


Figure S1. The deficiency of TRPV1 alleviates sperm damage in cryptorchid mice. (A) PAS staining of testes sections of sham group and cryptorchid group. P, pachytene spermatocyte; Z, zygotene spermatocyte; M, meiotic spermatocyte; rST, round spermatid; eST, elongating spermatid. (B) Representative IF images showing sham and cryptorchid cells stained with SYCP3 (red) and PNA (green).

Table S1: Primer sequences used for RT-qPCR

| Gene | Forward 5’-3’ | Reverse 5’-3’ |
| --- | --- | --- |
| *Trpv1* | TCTGCTGGAATCCTCGGGTGTAG | CATGCTGGTGTCTGTGGTACTGTAC |
| *Capn1* | CTTCCAAGACGAGGCCTTCC | GGGTGGCTCCATCCACGA |
| *Capn2* | ATCGAATGGAAGCGGCCC | AGCCAGGATTTCCTCATTCAA |
| *Bax* | TGCTAGCAAACTGGTGCTCA | CAGCCACCCTGGTCTTGGAT |
| *Aifm1* | CGAGCCCGTGGTATTCGA | CCATTGCTGGAACAAGTTGC |
| *Caspase 3* | TGTCATCTCGCTCTGGTACG | AAATGACCCCTTCATCACCA |
| *Map3k5* | AGGCCAAGGCGTTCATACTG | GAGCCATACTCGCTACTGCT |
| *Itpr1* | CGTTTTGAGTTTGAAGGCGTTT | CATCTTGCGCCAATTCCCG |
| *Fas* | GAACCTCCAGTCGAAACCA | GCTGTGTCTTGGATGCTGTCA |
| *β-actin* | GGTGGGAATGGGTCAGAAGG | GTACATGGCTGGGGTGTTGA |

Table S2: Primary and Secondary Antibodies

| Type | Antibody | Application | Dilution | Species | Manufacturer and Catalog Number |
| --- | --- | --- | --- | --- | --- |
| Primary | anti-TRPV1 antibody | WB | 1:1000 | Rabbit Polyclonal | Abcam, ab31895 |
|  | Calpain 1 Antibody | WB | 1:1000 | Rabbit monoclonal | Abcam, ab108400 |
|  | Calpain 2 Antibody | WB | 1:1000 | Rabbit monoclonal | Abcam, ab126600 |
|  | BAX Antibody | WB | 1:1000 | Rabbit monoclonal | Abcam, ab182733 |
|  | AIFM1 antibody | WB | 1:1000 | Rabbit Polyclonal | Affinity, DF7021 |
|  | Caspase 3 Antibody | WB | 1:1000 | Rabbit monoclonal | Abcam, ab238440 |
|  | MAP3K5 antibody | WB | 1:1000 | Rabbit Polyclonal | Affinity, AF6477 |
|  | ITPR1 antibody | WB | 1:1000 | Rabbit Polyclonal | Affinity, DF3000 |
|  | FAS antibody | WB | 1:1000 | Rabbit Polyclonal | Affinity, AF5342 |
|  | anti-β-actin antibody | WB | 1:10000 | Mouse monoclonal | Affinity, T0022 |
|  | anti-TRPV1 antibody | IF & IHC | 1:200 | Rabbit polyclonal | Alomone labs, ACC-030 |
|  | anti-SOX9 antibody | IF | 1:400 | Mouse polyclonal | Merck, AB5535-25UG |
|  | anti-PLZF antibody | IF | 1:50 | Mouse monoclonal | Santa Cruz, sc-28319 |
|  | anti-SCP3 antibody | IF | 1:400 | Mouse monoclonal | Abcam, ab97672 |
|  | anti-Phospho-Histone H2A.X rabbit monoclonal antibody | IF | 1:400 | Rabbit monoclonal | Cell Signaling, 9718 |
| Secondary | HRP-conjugated Affinipure Goat Anti-Mouse IgG | WB | 1:10000 | Goat Anti-Mouse IgG | Zhong Shan Jin Qiao, ZB-2305 |
|  | HRP-conjugated Affinipure Goat Anti-Rabbit IgG | WB | 1:10000 | Goat Anti-Rabbit IgG | Antiprotech, SA00001-2 |
|  | Alex Fluor 488-conjugated AffiniPure Donkey Anti-Mouse IgG | IF | 1:400 | Donkey Anti-Mouse IgG | Jackson Immuno Research, AB_2340846 |
|  | Cy™3 AffiniPure Donkey Anti-Rabbit IgG | IF | 1:400 | Donkey Anti-Rabbit IgG | Jackson Immuno Research, AB_2307443 |
|  | Goat Anti-Rabbit IgG, HRP Conjugated | IHC | 1:200 | Goat Anti-Rabbit IgG | Cwbio, CW0103S |
