# Supplementary material for: The involvement of TRPV1 in the apoptosis of spermatogenic cells in the testis of mice with cryptorchidism
Source: Cell Death Discov. 2025 Apr 3;11:135. doi: 10.1038/s41420-025-02447-3 (PMC11968804; doi:10.1038/s41420-025-02447-3)
Supplement: Supplementary file 2 — Original Western Blots [file 41420_2025_2447_MOESM2_ESM.pptx]

## Slide 1
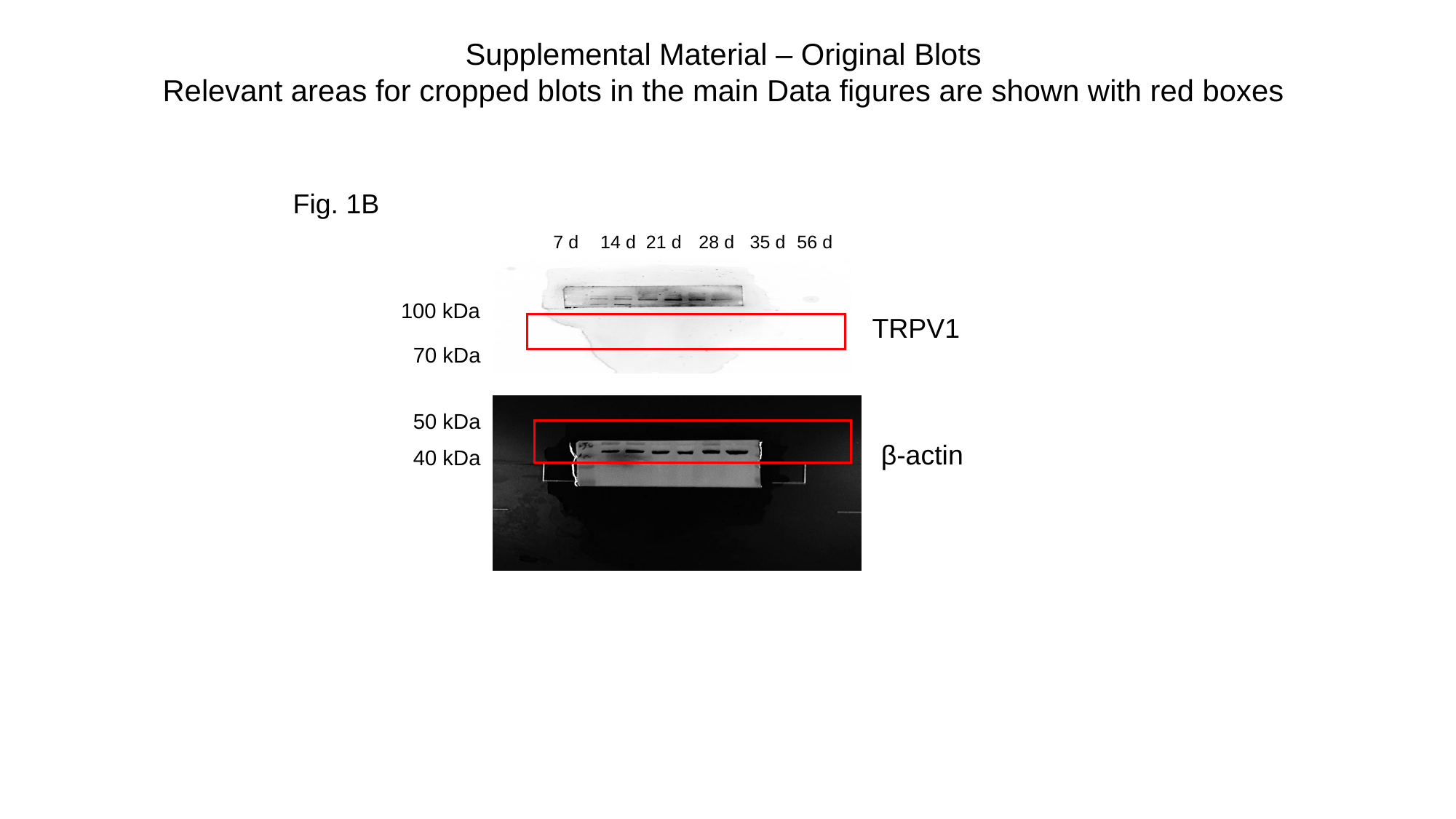

Supplemental Material – Original Blots
Relevant areas for cropped blots in the main Data figures are shown with red boxes
 Fig. 1B
7 d
14 d
21 d
28 d
35 d
56 d
100 kDa
TRPV1
70 kDa
50 kDa
β-actin
40 kDa

## Slide 2
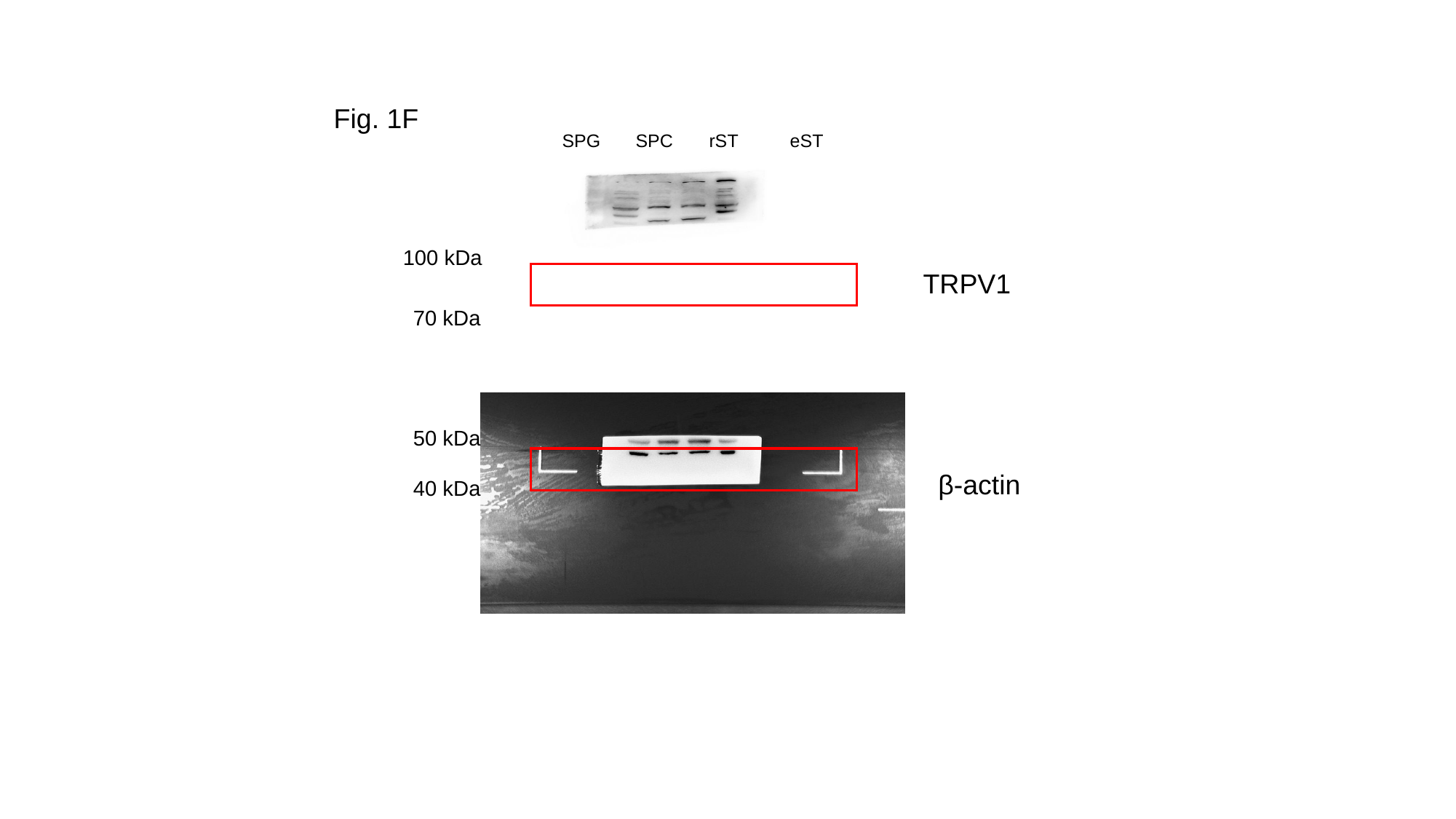

Fig. 1F
SPG
SPC
rST
eST
100 kDa
TRPV1
70 kDa
50 kDa
β-actin
40 kDa

## Slide 3
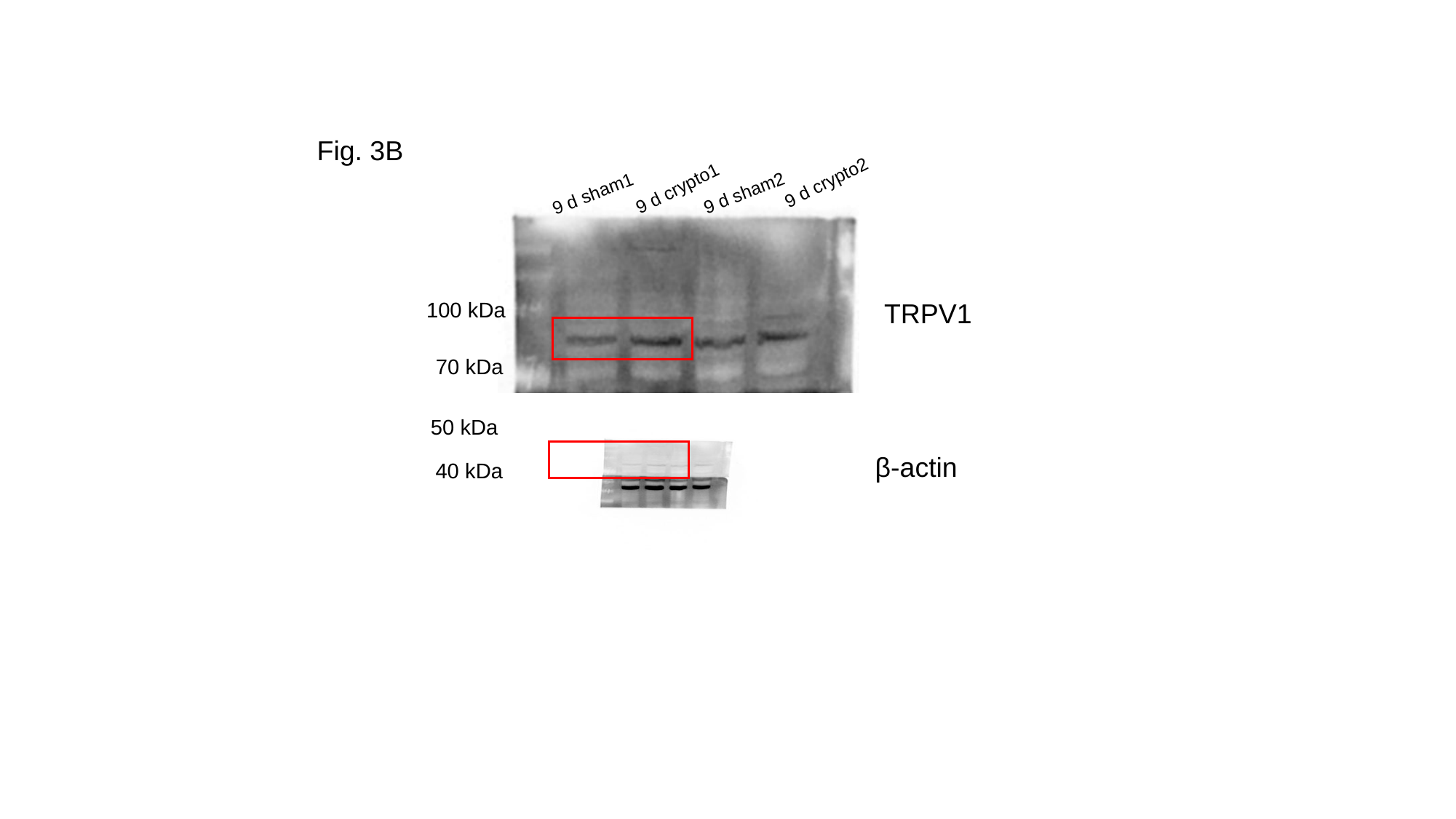

Fig. 3B
9 d crypto2
9 d crypto1
9 d sham2
9 d sham1
100 kDa
TRPV1
70 kDa
50 kDa
β-actin
40 kDa

## Slide 4
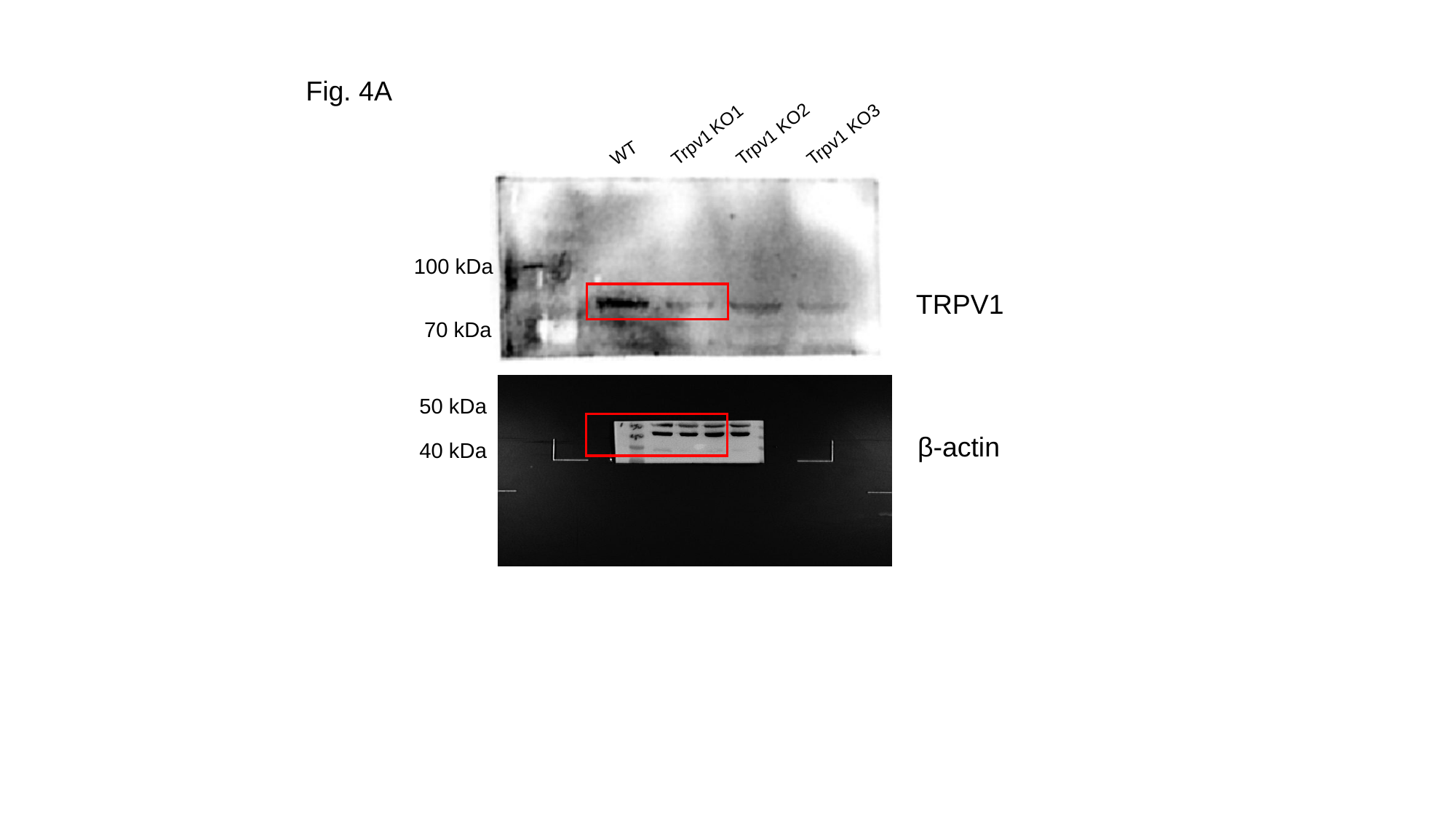

Fig. 4A
Trpv1 KO1
Trpv1 KO2
Trpv1 KO3
WT
100 kDa
TRPV1
70 kDa
50 kDa
β-actin
40 kDa

## Slide 5
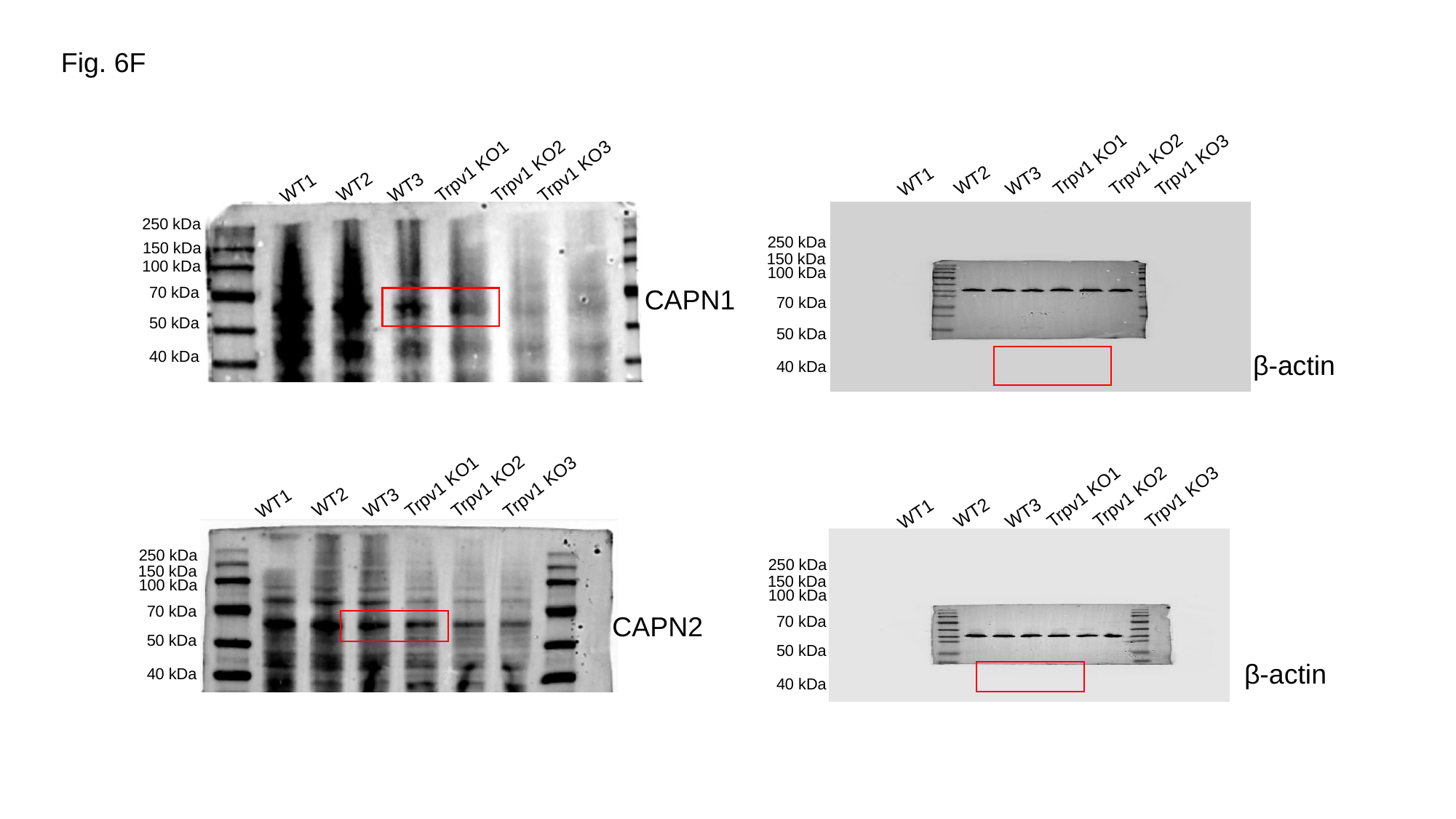

Fig. 6F
Trpv1 KO1
Trpv1 KO2
Trpv1 KO3
Trpv1 KO1
Trpv1 KO2
Trpv1 KO3
WT2
WT3
WT1
WT2
WT3
WT1
250 kDa
250 kDa
150 kDa
150 kDa
100 kDa
100 kDa
70 kDa
CAPN1
70 kDa
50 kDa
50 kDa
40 kDa
β-actin
40 kDa
Trpv1 KO1
Trpv1 KO2
Trpv1 KO3
Trpv1 KO1
Trpv1 KO2
Trpv1 KO3
WT2
WT3
WT1
WT2
WT3
WT1
250 kDa
250 kDa
150 kDa
150 kDa
100 kDa
100 kDa
70 kDa
CAPN2
70 kDa
50 kDa
50 kDa
β-actin
40 kDa
40 kDa

## Slide 6
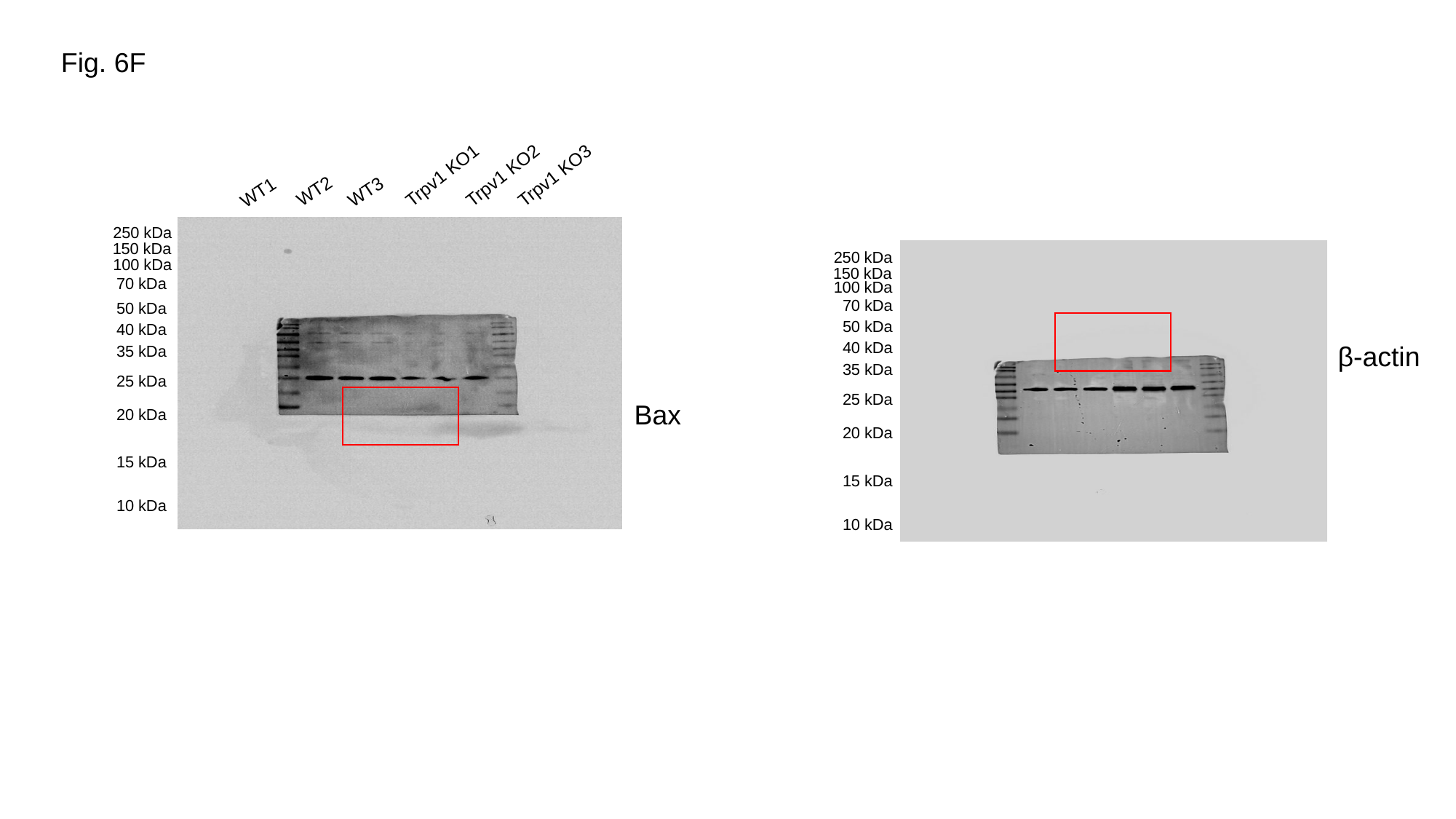

Fig. 6F
Trpv1 KO1
Trpv1 KO2
Trpv1 KO3
WT2
WT3
WT1
250 kDa
150 kDa
250 kDa
100 kDa
150 kDa
70 kDa
100 kDa
70 kDa
50 kDa
50 kDa
40 kDa
40 kDa
β-actin
35 kDa
35 kDa
25 kDa
25 kDa
Bax
20 kDa
20 kDa
15 kDa
15 kDa
10 kDa
10 kDa

## Slide 7
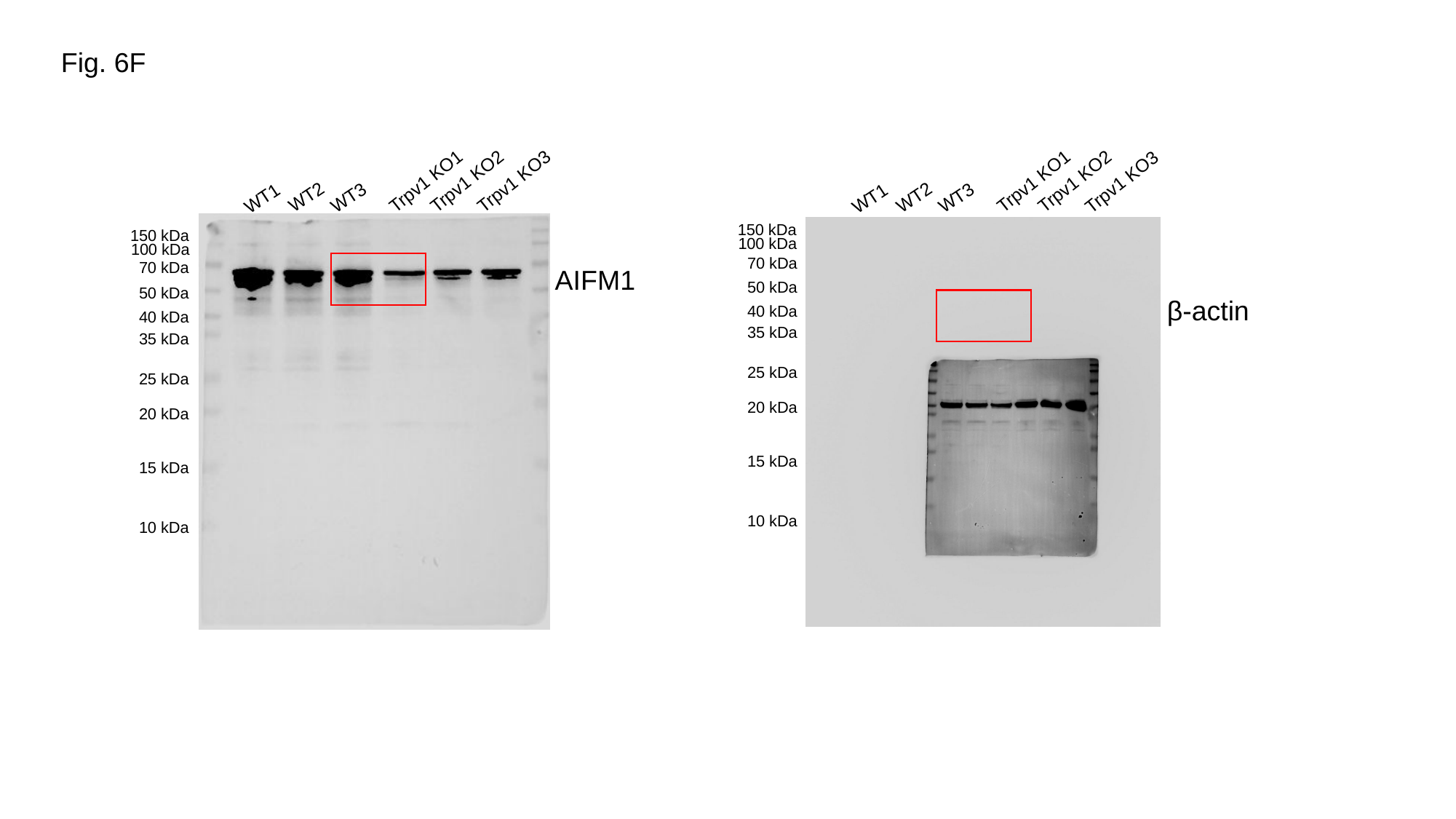

Fig. 6F
Trpv1 KO1
Trpv1 KO2
Trpv1 KO1
Trpv1 KO2
Trpv1 KO3
Trpv1 KO3
WT2
WT2
WT3
WT3
WT1
WT1
150 kDa
150 kDa
100 kDa
100 kDa
70 kDa
70 kDa
AIFM1
50 kDa
50 kDa
β-actin
40 kDa
40 kDa
35 kDa
35 kDa
25 kDa
25 kDa
20 kDa
20 kDa
15 kDa
15 kDa
10 kDa
10 kDa

## Slide 8
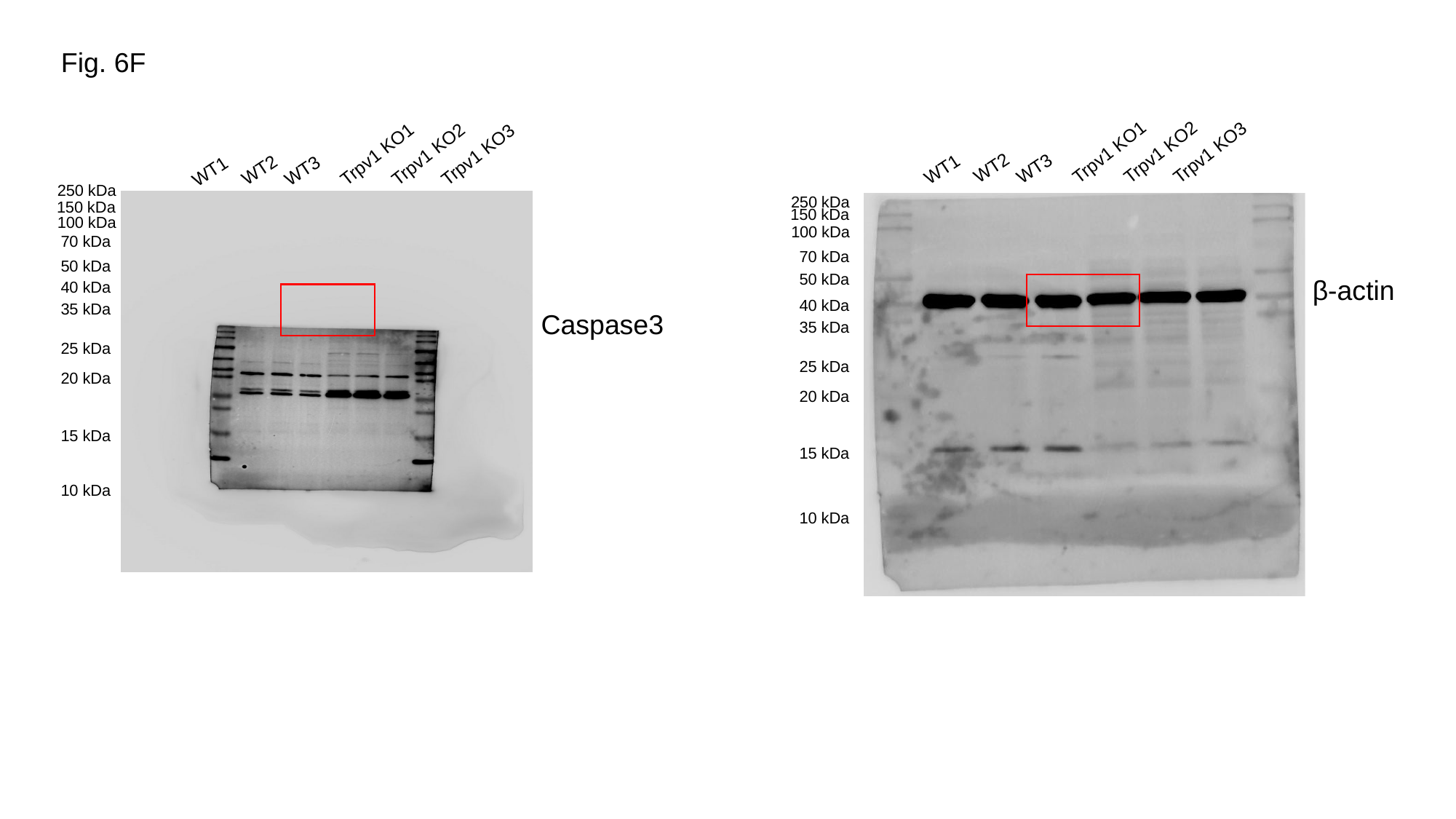

Fig. 6F
Trpv1 KO1
Trpv1 KO2
Trpv1 KO3
Trpv1 KO1
Trpv1 KO2
Trpv1 KO3
WT2
WT3
WT1
WT2
WT3
WT1
250 kDa
250 kDa
150 kDa
150 kDa
100 kDa
100 kDa
70 kDa
70 kDa
50 kDa
50 kDa
β-actin
40 kDa
40 kDa
35 kDa
Caspase3
35 kDa
25 kDa
25 kDa
20 kDa
20 kDa
15 kDa
15 kDa
10 kDa
10 kDa

## Slide 9
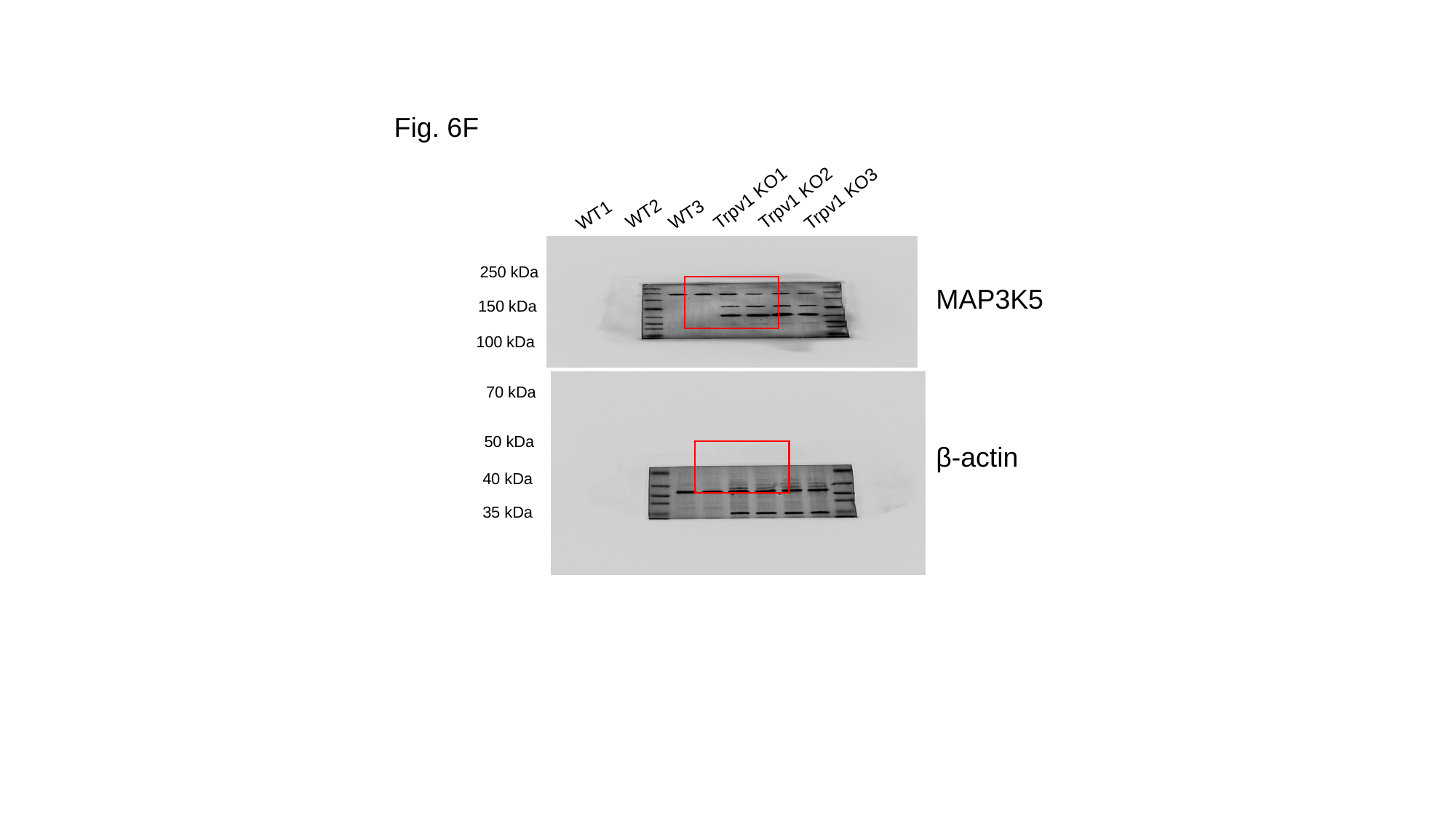

Fig. 6F
Trpv1 KO1
Trpv1 KO2
Trpv1 KO3
WT2
WT3
WT1
250 kDa
MAP3K5
150 kDa
100 kDa
70 kDa
50 kDa
β-actin
40 kDa
35 kDa

## Slide 10
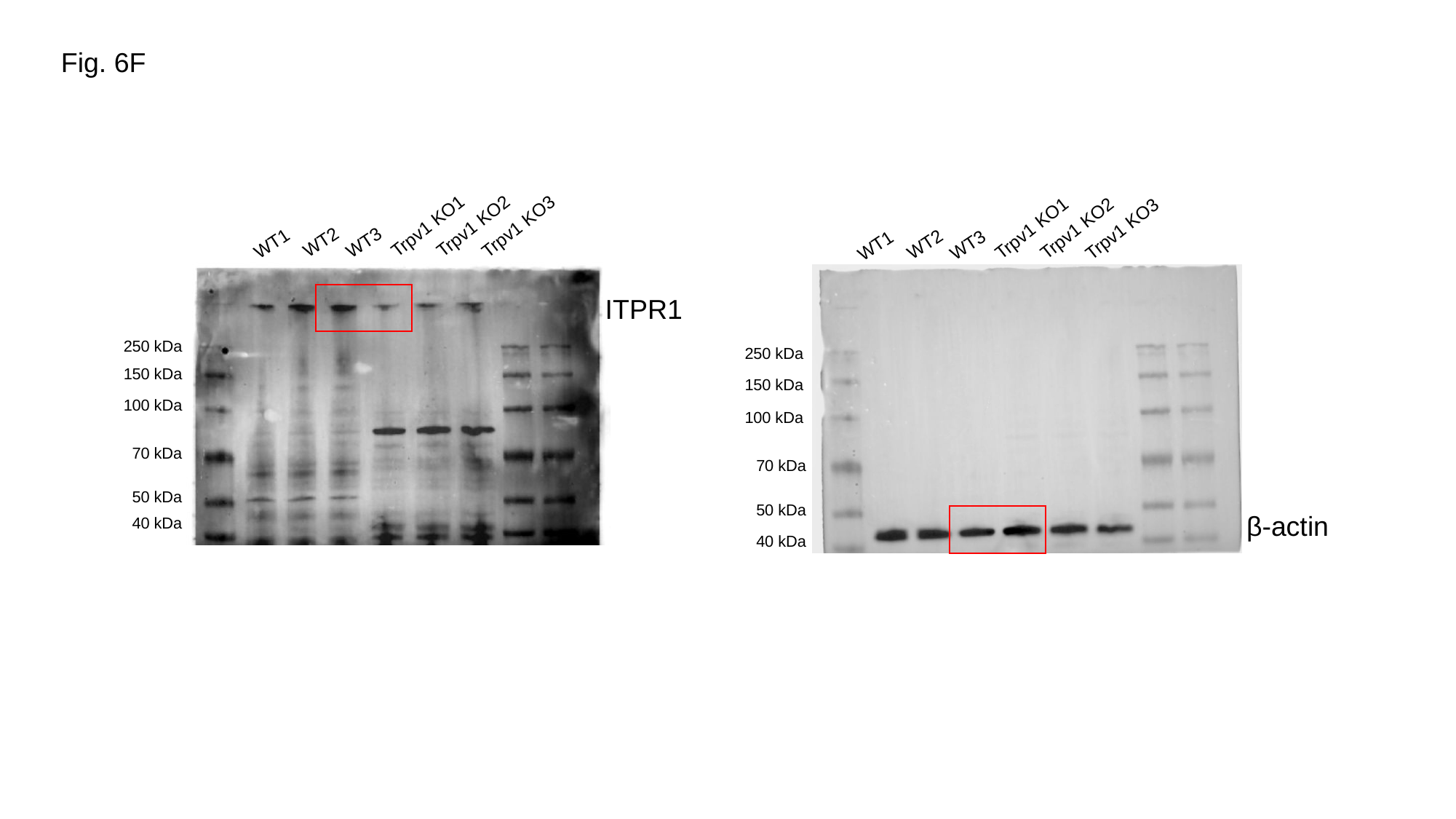

Fig. 6F
Trpv1 KO1
Trpv1 KO2
Trpv1 KO3
Trpv1 KO1
Trpv1 KO2
Trpv1 KO3
WT2
WT3
WT1
WT2
WT3
WT1
ITPR1
250 kDa
250 kDa
150 kDa
150 kDa
100 kDa
100 kDa
70 kDa
70 kDa
50 kDa
50 kDa
β-actin
40 kDa
40 kDa

## Slide 11
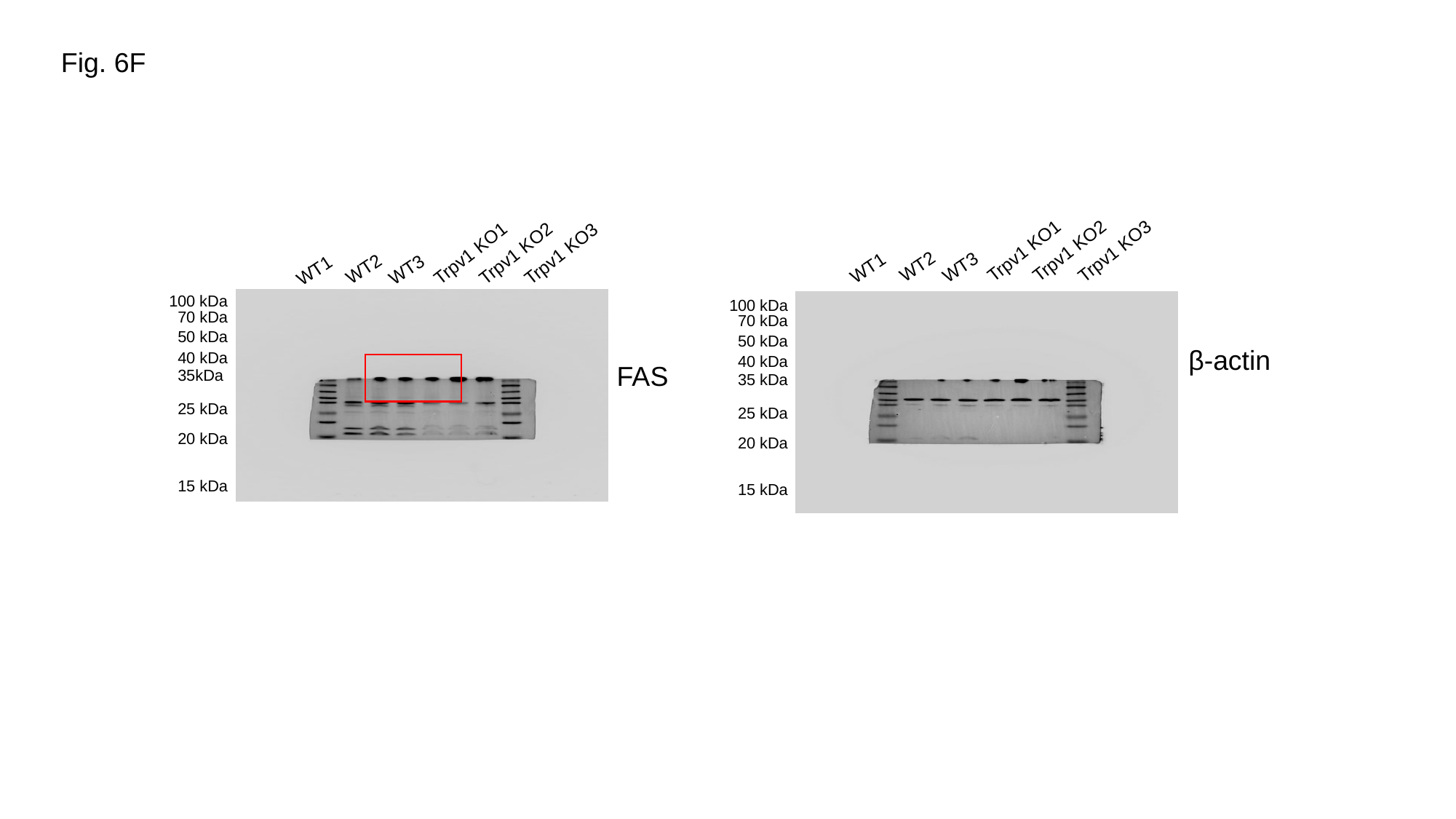

Fig. 6F
Trpv1 KO1
Trpv1 KO2
Trpv1 KO3
Trpv1 KO1
Trpv1 KO2
Trpv1 KO3
WT2
WT3
WT1
WT2
WT3
WT1
100 kDa
100 kDa
70 kDa
70 kDa
50 kDa
50 kDa
β-actin
40 kDa
40 kDa
FAS
35kDa
35 kDa
25 kDa
25 kDa
20 kDa
20 kDa
15 kDa
15 kDa
